# Depletion of Human Histone H1 Variants Uncovers Specific Roles in Gene Expression and Cell Growth

**DOI:** 10.1371/journal.pgen.1000227

**Published:** 2008-10-17

**Authors:** Mónica Sancho, Erika Diani, Miguel Beato, Albert Jordan

**Affiliations:** Centre de Regulació Genòmica (CRG-UPF), Barcelona, Spain; Fred Hutchinson Cancer Research Center, United States of America

## Abstract

At least six histone H1 variants exist in somatic mammalian cells that bind to the linker DNA and stabilize the nucleosome particle contributing to higher order chromatin compaction. In addition, H1 seems to be actively involved in the regulation of gene expression. However, it is not well known whether the different variants have distinct roles or if they regulate specific promoters. We have explored this by inducible shRNA-mediated knock-down of each of the H1 variants in a human breast cancer cell line. Rapid inhibition of each H1 variant was not compensated for by changes of expression of other variants. Microarray experiments have shown a different subset of genes to be altered in each H1 knock-down. Interestingly, H1.2 depletion caused specific effects such as a cell cycle G1-phase arrest, the repressed expression of a number of cell cycle genes, and decreased global nucleosome spacing. On its side, H1.4 depletion caused cell death in T47D cells, providing the first evidence of the essential role of an H1 variant for survival in a human cell type. Thus, specific phenotypes are observed in breast cancer cells depleted of individual histone H1 variants, supporting the theory that distinct roles exist for the linker histone variants.

## Author Summary

Eukaryotic DNA is packaged into chromatin through its association with histone proteins. The linker histone H1 sits at the base of the nucleosome near the DNA entry and exit sites to stabilize two full turns of DNA. In particular, histone H1 participates in nucleosome spacing and formation of the higher-order chromatin structure. In addition, H1 seems to be actively involved in the regulation of gene expression. Histone H1 in mammals is a family of closely related, single-gene encoded proteins, including five somatic subtypes (from H1.1 to H1.5) and a terminally differentiated expressed isoform (H1.0). It is not well known whether the different variants have distinct roles or if they regulate specific promoters. We have explored this by inducible knock-down of each of the H1 variants in breast cancer cells. A different subset of genes is altered in each H1 knock-down, and depletion has different effects on cell survival. Interestingly, H1.2 and H1.4 depletion specifically caused arrest of cell proliferation. Concomitant with this, H1.2 depletion caused decreased global nucleosome spacing and repressed expression of a number of cell cycle genes. Thus, specific phenotypes are observed in breast cancer cells depleted of individual histone H1 variants.

## Introduction

Eukaryotic DNA is packaged into chromatin through its association with histone proteins. Chromatin is composed of nucleosomes. The nucleosome core particle consists of 146 base pair units wrapped around a histone octamer consisting of two copies each of the core histone proteins H2A, H2B, H3 and H4. The linker histone H1 sits at the base of the nucleosome near the DNA entry and exit sites and is involved in the folding and stabilization of the 30 nm chromatin fiber [1],[2]. The amount of H1 per nucleosome is very variable, and the paradigm of one H1 per nucleosome is more the exception than the rule [3]. Histone H1 is a lysine-rich protein with a short basic N-terminal tail, a highly conserved central globular domain and a long positively-charged C-terminal tail. These tails are post-translationally modified, mostly by phosphorylation, but also by acetylation and methylation [4],[5]. CDK-dependent phosphorylation of H1 occurs progressively throughout the cell cycle, with a maximum during mitosis [6].

Histone H1 in vertebrates is a family of closely related, single-gene encoded proteins, showing much less evolutionary conservation than core histones. In mammals, five somatic subtypes (from H1.1 to H1.5), a terminally differentiated expressed isoform (H1.0), two tissue-specific variants (H1 testis and H1 oocyte) and a recently described, poorly characterized H1x variant have been identified [7]–[10].

Histone H1 participates in nucleosome positioning or spacing and formation of the higher-order chromatin structure. H1-containing chromatin is more resistant to nuclease digestion and shows strong inhibition of nucleosome sliding [11]. Consequently, H1 is seen as a structural component related to chromatin compaction and inaccessibility to transcription factors or RNA polymerase. Nonetheless, it has been suggested that histone H1 plays a more dynamic and gene-specific role, participating in activation or repression of gene expression. Previous studies on the effect of H1 depletion on global gene expression have reported changes in the expression of small groups of genes, instead of it affecting the vast majority of cellular genes [12]–[16]. Overexpression experiments have also contributed to challenge the concept of H1 as a general repressor of chromatin activity. In Xenopus laevis embryos, over-expression of the somatic H1 variant repressed oocyte- but not somatic-type 5S rRNA genes or other Pol III transcripts [17]. Overexpression of H1.0 and H1.2 isoforms increase both the basal and hormone-induced levels of the mouse mammary tumor virus (MMTV) reporter gene transcript [18].

Gene-specific effects of H1 might result from interactions with specific regulatory factors or DNA-binding proteins. This might also be the origin of reported specific functions for some H1 variants. H1.5 cooperates with the transcription factor MSX1 for inhibition of specific target promoters and myogenesis [19]. H1.4 is involved in a heterochromatinization process. H1.4 at Lys26 is deacetylated by SirT1 and subsequently methylated, facilitating recruitment of Polycomb complexes and HP1, whereas simultaneous phosphorylation of Ser27 blocks HP1 binding [20]–[23]. Recently, a novel H1.2 complex acting as a repressor of p53-mediated transcription has been isolated [24]. In addition, H1.2 has been involved in apoptosis induced by DNA double-strand breaks, acting as a cytochrome c-releasing factor that appears in the cytoplasm after X-ray irradiation [25].

A strong decrease in histone H1 has drastic consequences on the life span of some organisms [26]–[28]. However, single or double H1 variant knock-out mice have no apparent phenotype due to the compensatory up-regulation of other subtypes [29]. These reports have favored the thinking that H1 variants are redundant, lacking specific functions in chromatin organization or gene expression control. Knocking out additional subtypes cannot be compensated for fully by up-regulation of the remaining subtypes [30]. Nonetheless, triple null H1.2-H1.3-H1.5 mouse embryonic stem (ES) cells with a 50% global reduction in total H1 can be obtained [12]. The electrostatic effect of this reduction is apparently compensated for by a reduction in nucleosome repeat length (NRL), in line with a previously reported strong linear relationship between NRL and the H1/nucleosome ratio (reviewed in [3]), together with other mechanisms such as changes in core histone modifications. In this triple KO ES cell line, a very limited number of genes change their expression. These include imprinted genes regulated by DNA methylation, in which specific CpG regions are downmethylated in the absence of H1 [12].

We have previously described a complex role of the linker histone in the chromatin remodeling events taking place at the MMTV promoter in response to progestin. In vitro, histone H1 enhances transactivation in the simultaneous presence of the progesterone receptor (PR) and Nuclear Factor 1 (NF1). The first step following initial binding of PR to the exposed HREs is phosphorylation of histone H1. Then, PR recruits an ATP-dependant chromatin remodeling complex, which changes the structure of the nucleosomal core particles making its DNA more accessible for NF1 and additional PR binding. In a last step, H1 leaves the promoter to enable efficient transcription initiation [31],[32]. In this work, no attempt was made to define the role of individual H1 variants.

In order to further characterize in vivo the role of histone H1 variants and, in particular, to ascertain whether there is any functional specificity, we developed an inducible shRNA expressing system for the depletion of individual H1 variants in the human breast cancer cell line T47D. We achieved reliable specific inhibition for each variant, avoiding compensation effects and demonstrated significant differences among H1 variants, with regard to cell cycle progression and gene expression. On the one hand, depletion of H1.4 leads to cell death in T47D. This is the first time that it has been noted that one variant is essential for survival in a human cell type. On the other, we describe the involvement of H1.2 in cell cycle progression. Its inhibition caused a G1 arrest, defects in chromatin structure and changes in expression of specific genes linked to cell cycle.

## Materials and Methods

### Cell Lines and Culturing Conditions

H1 knock-down cell lines were established from T47D-MTVL cells (carrying one stably integrated copy of luciferase reporter gene driven by the MMTV promoter; [33]. These cell lines were grown in RPMI 1640 medium, supplemented with 10% FBS, 2 mM L-glutamine, 100 U/ml penicillin, and 100 µg/ml streptomycin. Doxycycline (Sigma) was added at 2.5 µg/ml when indicated. Along a 6-day treatment with Dox, cells were passaged at day 3. When indicated, serum-containing media was replaced with serum-free media at day 4 for growth arrest. Images of H1 KD cell lines grown in normal conditions were taken using an inverted Leica DMR microscope.

Other cell lines used were grown as follows: MCF-7 cell line was grown at 37°C with 5% CO_2_ in MEM medium containing 10% fetal bovine serum, 1% penicillin/streptomycin, 1% non-essential amino acids, 1% sodium pyruvate and 1% glutamine. MCF10A was grown at 37°C with 5% CO_2_ in F-12 MEM medium containing 10% fetal bovine serum, 1% penicillin/streptomycin, 10 µl of insulin, 10^−8^ M cholera toxin, 0.5 µg/ml of hydrocortisone and 10 ng/ml of EGF. HEK 293T was grown at 37°C with 5% CO_2_ in Dulbecco's modified Eagle medium (DMEM) containing 10% fetal bovine serum, 1% glutamine and 1% penicillin/streptomycin. HeLa was grown at 37°C with 5% CO_2_ in DMEM medium containing 10% fetal bovine serum and 1% penicillin/streptomycin.

### shRNA Cloning, Virus Production, and Cell Infections

Plasmids for the lentivirus vector-mediated drug-inducible RNA interference system (pLVTHM, ptTR-KRAB-Red, pCMC-R8.91 and pMD.G) were provided by D. Trono (University of Geneva) [34]. The 64-mer oligonucleotides for histone H1 shRNA cloning into MluI/ClaI-digested pLVTHM were designed, annealed and phosphorylated as communicated by D. Trono (http://tronolab.epfl.ch/). Oligonucleotides have the following general structure: 5′-CGCGTCCCC-**N19** TTCAAGAGA-**rcN19**-TTTTTGGAAAT-3′ and 5′-AGGGG-**N19**-AAGTTCTCT-**rcN19**-AAAAACCTTTAGC-3′, being N19 the specific target sequence for each H1 variant and rcN19 its reverse complementary sequence. The corresponding 19-mer gene-specific target sequences for interference were designed manually following standard rules (AAN19, GC % 30–70). Target sequences (N19) are CGCTGACTCGCAGATCAAG for H1.0, AGAGCGTAGCGGAGTTTCT for H1.2, CTGCCAAGAGTCCAGCTAA for H1.3, GAAGAGCGCCAAGAAGACC for H1.4 and GGCAACTAAGAAGGCTGCC for H1.5.

For the production of viral particles containing the HIV-derived vectors, 2.5×10^6^ HEK 293T cells (Clontech) were transfected with plasmids ptTR-KRAB-Red, pLVTHM-*shH1.n* or pEV833-*HA-H1.n* (10 µg), pCMV-R8.91 (6.5 µg) and pMD.G (3.5 µg) in 10 cm dishes using calcium phosphate. Medium was collected every 24 hours for 2 days and centrifuged 1h30min at 26,000 rpm at 4°C in a sucrose gradient to concentrate viruses. Pellet containing viral particles was dissolved in medium and used for cell infection. Cells were infected using the spinoculation method, i.e. plates were centrifuged at 1,200× g for 2 h at 25°C.

Initially, a cell line expressing the Dox-responsive KRAB repressor and RedFP (ptTR-KRAB-Red) was generated. Then, this cell line was infected with viruses for expression of the different H1 variants shRNAs (pLVTHM). The inducible knocked-down cell lines were sorted in a FACSvantageSE (Becton Dickinson) for RedFP-positive and GFP-positive fluorescence after 3 days of Dox treatment. Then cells were amplified in the absence of Dox until an experiment was performed.

### Stable Expression of HA-Tagged H1 Variants

Human histone H1 variants were PCR-amplified from genomic DNA and cloned into pCDNA4-HA vector provided by D. Reinberg's group (NYU Medical School). The complete H1-HA cassette was cloned into the lentiviral expression vector pEV833 provided by E. Verdin (Gladstone Institutes) upstream an IRES-GFP cassette. Virus production and infections with pEV833-derived lentivirus were performed as described above. shRNA- resistant H1.2 and H1.4 were created by site-directed mutagenesis with QuikChange Mutagenesis Kit (Stratagene). The introduced changes (in lower case: AGAaCGgtcCGGcGTTagT for H1.2, and GAAatctGCgAAGAAGACC for H1.4) did not alter the amino acidic sequence.

### Production of H1 Variant Specific Antibodies

The N-terminal domain of human H1 variants H1.1 to H1.5 was used to design peptides for production of subtype-specific antibodies (Figure S1). Peptides were synthesized and conjugated to a carrier protein, Keyhole Limpet Hemocyanin (KLH), in the Proteomics Facility of the Pompeu Fabra University. Each of two rabbits was injected with 200 µg of peptide for each H1 variant, at two weeks intervals, for a total of 4 immunizations. Sera were collected and analyzed a week after last immunization. Antibodies were purified in a HiTrap Protein A HP column (Amersham Biosciences) according to manufacturer instructions and some are available through Abcam Ltd. (Cambridge, UK): H1.1 (ab17584), H1.3 (ab24174) and H1.5 (ab24175). A second generation of antibodies (including H1.2 ab17677 and H1.5 ab18208) was produced in collaboration with Abcam starting from two-branched peptides 2×(specific peptide)-Lys-Cys as reported elsewhere [35].

### Protein Extraction, Gel Electrophoresis, Immunoblotting, and Other Antibody-Based Methods

Histone H1 was purified by 5% perchloric acid lysis for 1 hour at 4°C. Soluble acid proteins were precipitated with 30% trichloroacetic acid over night at 4°C, washed twice with 0.5 ml of acetone and reconstituted in water. Whole cell extracts were obtained lysing cells in Tris-HCl (pH 7.4) 25 mM, EDTA 1 mM, EGTA 1 mM and SDS 1% plus protease and phosphatase inhibitors. Protein concentration was determined in both cases by Micro BCA protein assay (Pierce). Lysates or purified proteins were subjected to 12% SDS-PAGE, transferred to a nitrocellulose membrane, blocked with 5% non-fat milk for 1 hour, incubated with primary antibodies over night at 4°C and secondary antibodies conjugated to peroxidase for 1 hour at room temperature. Bands were visualized by chemiluminiscence using ECL system (Amersham). Quantifications were performed with Image Gauge (FujiFilm) software.

The anti H1.0 (ab11079), H1 phospho-T146 (ab3596), HA-tag (ab9110) and CDC2 (ab18) antibodies were from Abcam; anti tubulin was from Sigma; anti CDK2 (m2), CDK4 (c22), CCND1, p27 (c19), p16 (h156) and p21 (c19) antibodies were from Santa Cruz; phospho-Rb (Ser 608) was from Cell Signalling.

Immunofluorescence and chromatin immunoprecipitation (ChIP) assays with the HA-tag antibody were performed as described previously [36]. CDKs were immunoprecpitated from total cell extracts and its kinase activity measured on Rb or H1 protein substrates as described elsewhere [37],[38].

Recombinant human H1 subtypes were purchased from Alexis Biochemicals (produced in *E. coli* bacteria), or provided by N. Happel (University of Göttingen) (produced in yeast). Recombinant Rb fragment 792–925 was kindly provided by O. Bachs (IDIBABS-UB).

### Cell Cycle Analysis

Cells were washed with cold PBS 1×, fixed in 70% ethanol and stained with Analysis solution: 3% Ribonuclease A (Sigma) 10 mg/ml, 3% solution A (38 mM sodium citrate, 500 µg/ml propidium iodide) in PBS 1×. Samples were analyzed using a FACS Calibur machine (Becton Dickinson), CellQuest analysis software and ModFit program.

### MNase Digestion

Pellet of cells growing in rich medium and treated or not for 6 days with Dox was dissolved in buffer A (10 mM Tris-HCl pH 7.4, 10 mM NaCl, 3 mM MgCl_2_, 0.3 M sucrose and 0.2 mM PMSF) plus 0.2% of NP40 and incubated for 10 min at 4°C. Nuclei were obtained after centrifugation and digested with 2.5 units of Mnase (Worthington) per 4 million of nuclei for 25 min at room temperature in buffer A plus 10 mM CaCl_2_. DNA was purified through a Qiagen column and run on 1.2% agarose gel.

### RNA Extraction and RT-qPCR

Total RNA was extracted using the RNAsy Kit (Qiagen). The quality of the RNA was analyzed using the Agilent Bioanalyzer 2100 and the RNA 6000 LabChip Kit (Agilent) with the Eukaryote Total RNA Nano Assay. cDNA was generated from 100 ng of RNA using Superscript First Strand Synthesis System (Invitrogen). Gene products were analyzed by qPCR using SYBR Green Mix (Roche) and specific oligonucleotides in a Roche 480 Lightcycler. Each value was corrected by human GAPDH and expressed as relative units. Each experiment was performed in triplicate. Gene-specific oligonucleotide sequences are available on request.

### SuperArray RT-qPCR and Microarrays

The RT Profiler PCR Array (APHS-020) from SuperArray Biosciences Corporation is a 96-well plate containing primers for 84 cell cycle related genes, plus 5 housekeeping genes and 3 RNA and PCR quality controls. The system also includes an instrument-specific master mix and an optimized first strand synthesis kit. First strand synthesis and qPCR was performed following instructions from manufacturer and run in a Roche 480 Lightcycler.

Procedures for microarray hybridization and data analysis are described elsewhere [36],[39] and detailed in the Text S1.

Microarray data is available at GEO with accession numbers GSE11294 and GSE12299 (http://www.ncbi.nlm.nih.gov/geo/index.cgi).

## Results

### Inducible RNA Interference-Mediated Depletion of Histone H1 Variants in Human Breast Cancer Cells

In order to explore the specificity of the different H1 subtypes in gene expression control in breast cancer cells, we generated T47D-derived stable cell lines with reduced expression of each of the H1 variants specifically (H1.0 to H1.5). H1.1 was left out as it is not expressed in T47D cells (Figure S1). We used an inducible shRNA expression system based on a TeT-On strategy [34]. shRNAs targeting divergent gene regions were designed to specifically inhibit the H1.0, H1.2, H1.3, H1.4 and H1.5 variants of the linker histone and cloned in the lentivirus vector pLVTHM. Knocked-down cell lines were generated first by infecting the regulator vector that expresses a RedFP marker and a repressor TetR-KRAB fusion protein, then the vector codifying for the shRNA and reporter GFP. Upon doxycycline (Dox) addition, derepression of GFP and shRNA occurs and the targeted gene is knocked down. Cells simultaneously expressing RedFP and GFP upon Dox addition were selected by cell sorting. A control cell line was generated by infection with the empty pLVTHM vector.

**Figure 1:**
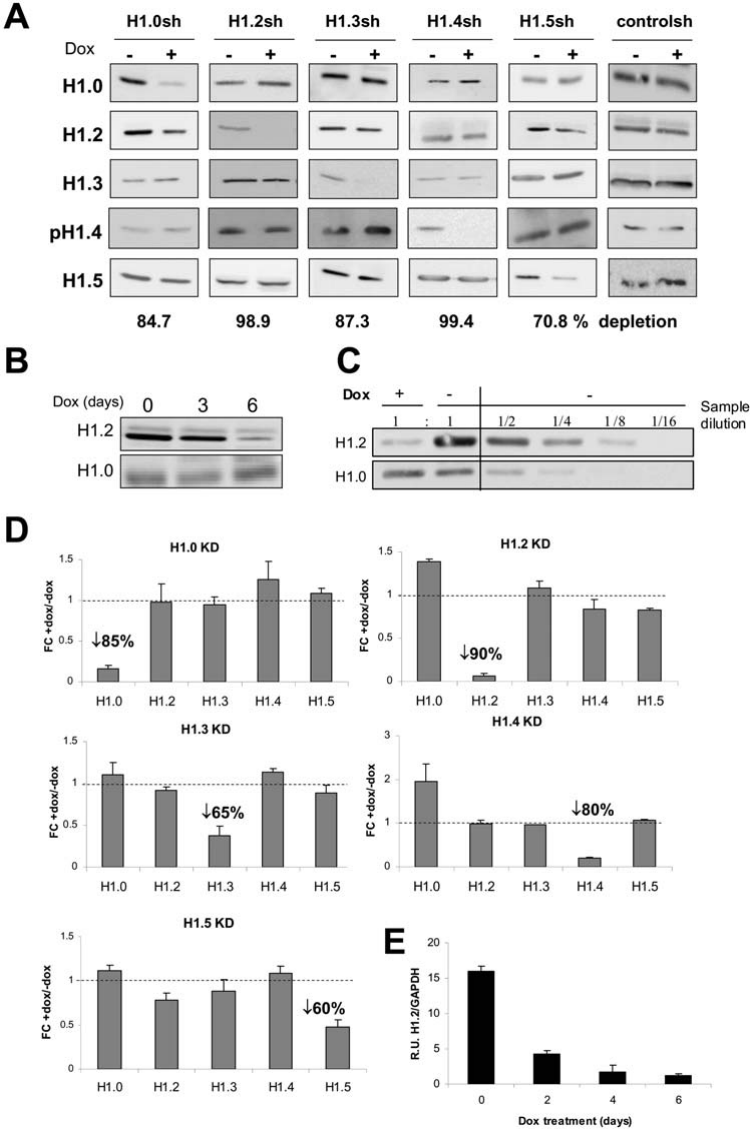
Inducible RNA interference-mediated knock-down of histone H1 variants in human breast cancer cells. T47D derivative cells stably infected with the lentiviral inducible system for the expression of shRNAs against each of the human histone H1 variants indicated were treated for 6 days with Dox or left untreated. (A) H1 depletion was tested by Western blot with isoform-specific antibodies on histone H1 preparations obtained by acid extraction. H1 phospho-T146 antibody was used to detect H1.4 (see Figure S1 for a characterization). The percentage of specific H1 isoform depletion upon quantification by band densitometry is shown underneath. (B) Optimal histone H1 depletion is achieved after 6 days of Dox treatment. Western blot analysis of H1.2 depletion in a Dox treatment time-curve. (C) Relative quantification of H1.2 depletion upon Dox treatment. Serial dilutions of the H1 sample extracted from non-treated cells were compared to a Dox-treated sample in a Western blot using anti H1.2 antibody. This comparison shows that H1.2 was reduced 8-fold. (D) Inhibition of H1 expression was tested by RT followed by real-time PCR (qPCR) with oligonucleotides specific for the different human H1 variant genes. Data is expressed as fold change (FC) of H1 gene expression corrected by GAPDH in response to Dox compared to untreated. The values represent the mean and SD of a representative experiment performed in triplicate. (E) The effect of H1 shRNAs is seen earlier when expression of the H1 mRNA is followed by RT-PCR. This example shows H1.2 mRNA reduction along a Dox time-course.

Depletion of the different H1 variants was analyzed by Western blot with specific antibodies (See Materials and Methods and Figure S1). H1.4 was detected with a H1 phospho-T146 antibody characterized in Figure S1. Western blot analysis of H1-extracted sample from each knocked-down cell line revealed a consistent and specific reduction of every H1 variant (Figure 1A). No significant compensation effects by other H1 subtypes were observed at protein level, but a H1.0 increase after H1.4 depletion. Efficient depletion was achieved after six days of treatment with Dox (Figure 1B). Relative quantification of H1.2 inhibition by Western blot showed a 8-fold decrease in protein level (Figure 1C). Depletion of other isoforms oscillated between 70 and 99% (Figure 1A). Electrospray mass spectrometry of H1 preparations from all Dox-treated cell lines also showed specific reduction of each H1 subtype identified by its differential molecular weight (data not shown).

The knock-down effect was also measured by reverse transcription and real-time PCR (RT-qPCR) analysis of H1 variants gene expression before and after Dox treatment for all the cell lines generated (Figure 1D). This showed specific reduction of the targeted gene (between 60% and 90% depending on the variant) and some increase in H1.0 mRNA in both H1.2 and H1.4 knocked-down cell lines. Significant reduction of H1 variants transcripts due to shRNA expression occurred 2 days after Dox addition (Figure 1E), earlier than the apparent reduction of H1 proteins.

### Effect of H1 Variants Depletion on Cell Proliferation and Cell Cycle Progression

**Figure 2:**
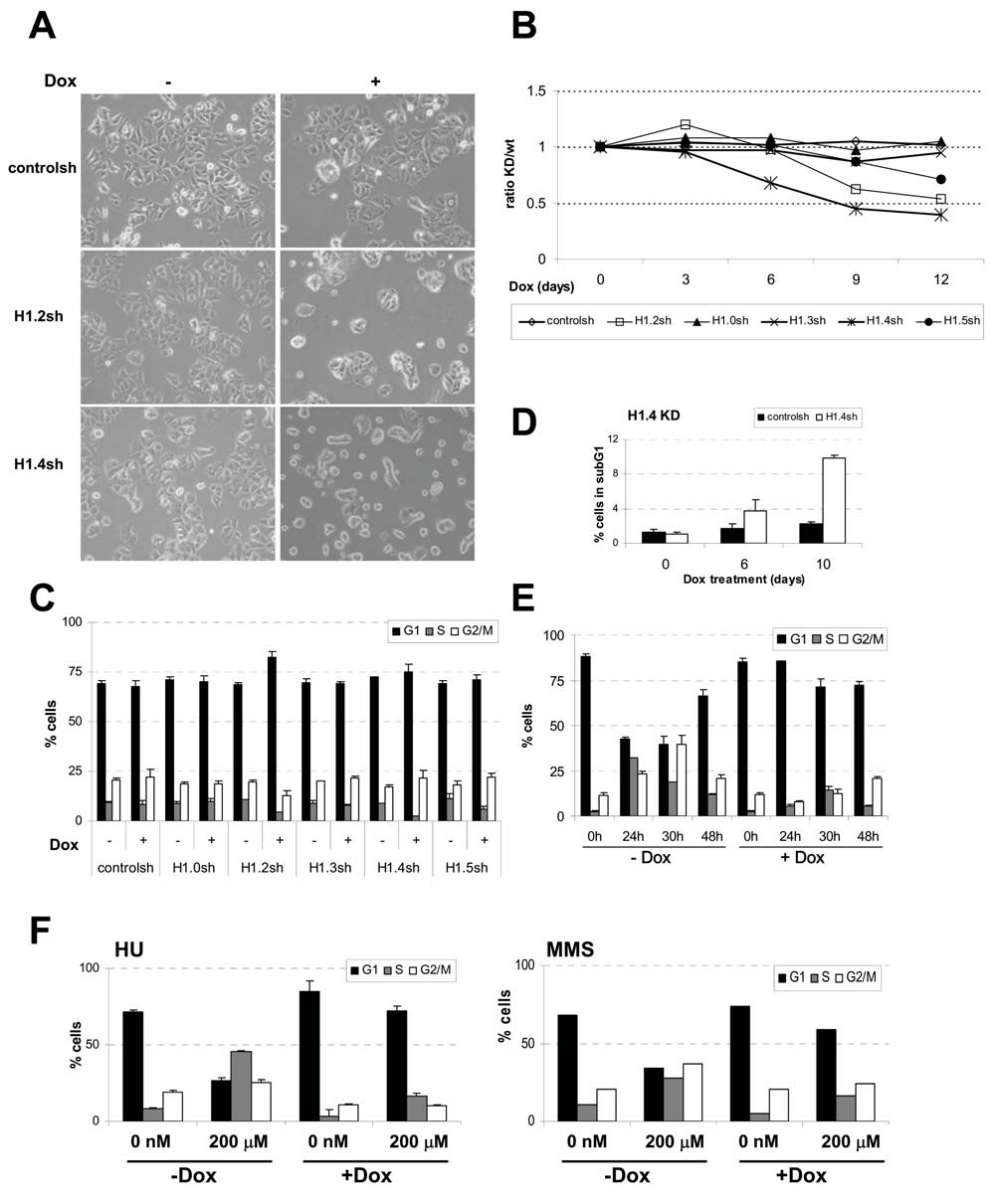
Inhibition of H1 variants causes different effects on cell proliferation and cell cycle progression. (A) T47D cells infected with the inducible shRNA-expression system against H1.2, H1.4 or empty plasmid were treated for 6 days with Dox or left untreated and observed at the optical microscopy. (B) In order to measure the effect of H1 depletion on cell proliferation, each H1 variant knock-down cell line (RFP and GFP-positive) was mixed 1∶1 with parental T47D cells (RFP and GFP-negative) and treated with Dox. Every three days, cells were split and the percentage of RFP/GFP-positive cells measured by FACS. Data is expressed as ratio of RFP/GFP-positive (KD) versus RFP/GFP-negative (wild-type) cells along time. Data corresponds to a representative experiment performed in duplicate. (C) Cell cycle profile after propidium iodide (PI) staining of H1 variant knock-down cell lines grown for six days in the presence or absence of Dox. Data is expressed as percentage of cells in G1, S and G2/M cell cycle phases. The values represent the mean and SD of a representative experiment performed in triplicate. (D) H1.4 knocked-down and control cells grown in the presence of Dox for 6 and 10 days were analyzed by FACS after PI staining to measure the percentage of cells in subG1phase as indicative of cell death. (E) H1.2 KD cells grown for six days in the presence or absence of Dox and without serum for the last two days, were treated with serum and the cell cycle profile was analyzed at time points indicated. (F) Cell cycle profile of H1.2 KD cells in response to treatment with different inhibitors. Cells were treated or not with Dox for 6 days. Hydroxyurea (HU) or methyl methanosulfonate (MMS) were added at the indicated concentrations 12 h before analysis of the cell cycle profile by FACS measurement of PI-stained cells.

Upon Dox treatment, we observed differences in growth rate among H1 variant knock-downs, in particular, H1.2 and H1.4 depleted cells failed to reach confluency (Figure 2A) and exhibited a slower growth rate. To quantify this we mixed, at a 1∶1 ratio, each of the shRNA expressing cell lines (RedFP and GFP-positive upon Dox treatment) with parental T47D cells (RedFP and GFP-negative), and we monitored by FACS the proportion between the two populations over time in culture in the presence of Dox (Figure 2B). Slow progression of H1.4 knocked-down cells was seen at six days after Dox addition and of H1.2 depleted cells at day 9. At day 12, H1.5 depleted cells were also less abundant than the parental ones. In addition, at day 6, H1.4 cells had changed morphology towards a necrotic phenotype (Figure 2A and Figure S2).

To understand the causes of these proliferation defects, the cell cycle profile of the different cell lines was examined by FACS analysis of propidium iodide-stained cells six days after Dox addition and compared to untreated cells. The knocked-down cell lines for H1.0 and H1.3 did not show any significant difference in the cell cycle profile in comparison to uninduced cells or to the control cell line. On the other hand, both H1.2 and H1.4 depleted cells exhibited a dramatic reduction in the S phase cell population (Figure 2C and Table S1). H1.5 depletion caused a slight decrease of S phase. Interestingly, in the H1.2 knock-down, an increase in the G1 peak over controls and a concomitant decrease in G2 peak were observed. That is, depletion of H1.2 induced G1 arrest. We cannot discard that complete depletion of H1.0, H1.3 or H1.5 could have different effects.

### H1.4 Is Required for Survival of T47D Cells

In the H1.4 knock-down, a small increase of G2 and almost no change in G1 was observed. Although the cell cycle pattern was not very dramatic, the increase in the subG1 peak indicates a high level of mortality in the H1.4 depleted cells (Figure 2D), in accordance with the cell growth experiments. Experiments performed to measure apoptotic cells (such as formation of apoptotic DNA ladder, anexin V binding or TUNEL assays) have failed to find differences between H1.4-depleted and control cells, indicating that cell death is not mediated by apoptosis (data not shown). Based on these results, we conclude that H1.4 knocked-down cells show a cell death phenotype, probably by necrosis, although more accurate analysis needs to be done. The deleterious effect of H1.4 depletion in T47D cells was rescued by transient transfection of a plasmid expressing recombinant shRNA-resistant H1.4 (Figure S2), discarding off-target effects.

### H1.2 Depletion Causes Cell Cycle Arrest in G1

To further characterize the G1 arrest phenotype of H1.2-depleted cells, we studied the cell cycle progression after recovering from a serum-starvation block in G1. H1.2 knock-down (KD) cells treated or not with Dox for 6 days were serum-starved for the last two days. After serum addition, the cell cycle profile was determined at 24, 30 and 48 h (Figure 2E and Table S1). Dox untreated cells progressed along the cycle, with a maximum of cells in S phase at 24 h, in G2/M at 30 h, and in G1 at 48 h. Dox-treated cells remained arrested in G1-phase, with little increase in cells in S-phase at 30 h after serum addition. This indicated that the G1 block caused by H1.2 depletion is strong and very few cells are able to escape and progress through the cell cycle.

Hydroxyurea (HU) and methyl methanosulfonate (MMS) are damaging agents that block cycling cells in S and S/G2 phase, respectively. We investigated how these drugs affect the cell cycle profile of ±Dox-treated H1.2 and H1.4 KD cells. While the cell cycle profile of H1.4 KD in response to HU or MMS was practically indistinguishable in the presence or absence of Dox (data not shown), H1.2 KD cells treated with Dox remained accumulated in G1 whether or not HU or MMS was present (Figure 2F). Taken together, our results indicated that H1.2 depletion causes a strong G1 arrest phenotype and that H1.4 KD cell cycle is not greatly affected in the few surviving cells.

### G1 Arrest in H1.2 Depleted Cells Is Reverted Specifically by Expression of the Targeted Protein

**Figure 3:**
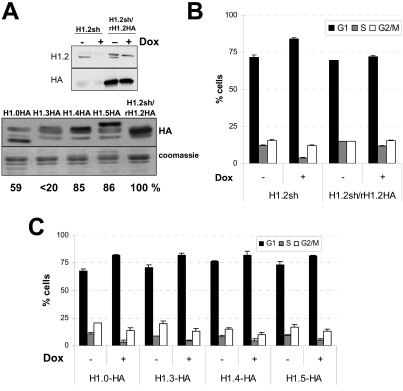
G1 arrest in H1.2 depleted cells is reverted specifically by expression of the targeted protein. (A) Expression of HA-tagged H1 variants in H1.2 knocked-down cells. shRNA-resistant H1.2 and the rest of H1 variants, fused to HA peptide at their C-termini, were introduced by lentiviral infection into shH1.2 cells. Expression was measured with an anti-HA or H1 variant-specific antibodies by Western blot. Cells were treated with Dox for 6 days when indicated. Coomassie staining of H1 preparations is shown as a loading standard. The degree of expression of each HA-tagged variant in comparison to rH1.2-HA (100%) is shown. H1.3-HA could not be quantified precisely. (B) G1 arrest in H1.2 knocked-down cells is reverted by stable expression of recombinant HA-tagged, shRNA-resistant H1.2, (C) but not by expression of other H1 variants. The values represent the mean and SD of the percentage of cells in each of the cell cycle phases in H1.2 KD cells stably expressing the different H1 recombinant variants, treated or not with Dox for 6 days. Representative experiments performed in triplicate are shown.

In order to demonstrate that the cell cycle arrest was specifically due to the depletion of the H1.2 variant, we stably integrated into the H1.2 KD cell line a lentiviral vector for the expression of an shRNA-resistant H1.2-encoding gene C-terminally fused to the HA peptide (rH1.2-HA). The levels of rH1.2-HA protein were similar to the endogenous histone variant as shown by Western blot analysis (Figure 3A). Cell cycle analysis demonstrated that the G1 arrest observed in the H1.2 depleted cells was reversed when the rH1.2-HA protein was present (Figure 3B).

Moreover, to exclude that the phenotype was not due to a reduction in the total H1 content, we performed the experiment stably expressing ectopically each of the other HA-tagged H1 variants in the inducible H1.2 KD cell line. Incorporation of recombinant HA-tagged H1 variants into chromatin was tested by several means, including Western blot of H1 extracted from chromatin, immunofluorescence and chromatin immunoprecipitation (Figure S3). Expression of H1.4-HA and H1.5-HA was comparable to rH1.2-HA, H1.0-HA level was sensibly lower, and H1.3-HA approximately 10-fold inferior (Figure 3A). As Figure 3C shows, none of the other variants was able to reverse the G1 arrest caused by the depletion of H1.2. Taken together, this indicates that H1.2 variant is specifically required for normal cell cycle progression and its depletion causes cells to accumulate in G1 phase. Complementation results with H1.3-HA and, probably, H1.0-HA are not definitive due to its lower expression levels.

### Variant-Specific Effects of H1 Depletion in Cell Progression Are Not Restricted to T47D Cells

**Figure 4:**
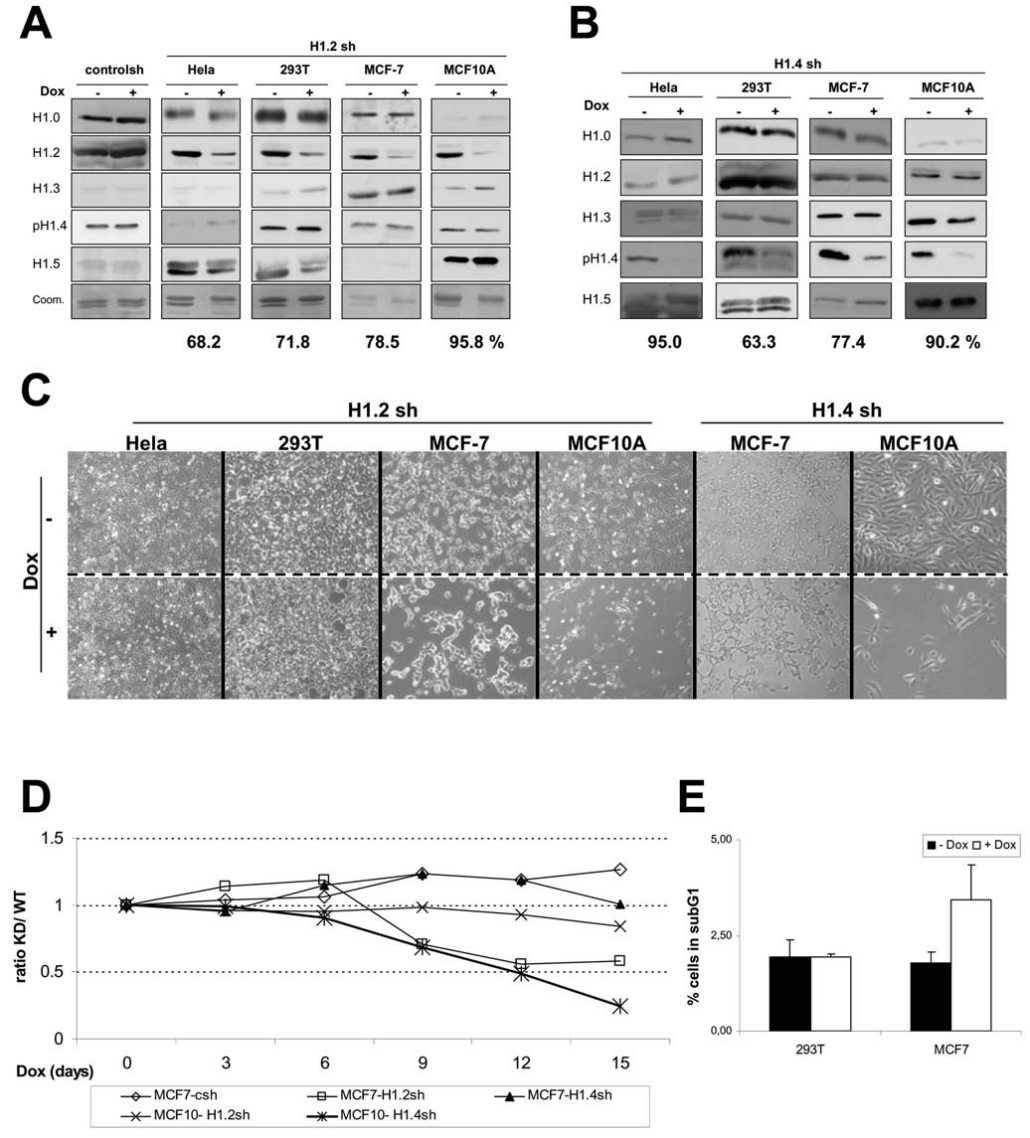
Variant-specific effects of H1 depletion in cell progression are not restricted to T47D cells. HeLa, 293T, MCF7 and MCF10A cells were infected with the inducible shRNA-expression system to inhibit expression of (A) H1.2 or (B) H1.4, and the efficacy of linker histone depletion was tested in Western blot with specific antibodies. 293T cells infected with the empty shRNA-expression vector are shown as control. The percentage of H1.2 (A) or H1.4 (B) depletion upon quantification by band densitometry is shown underneath. Coomassie staining of H1 preparation is shown for each H1.2 KD cell line, were depletion of the lower band corresponding to H1.2 in response to Dox can be observed. (C) Cell lines indicated infected with the inducible shRNA-expression system against H1.2 or H1.4 were treated for 6 days with Dox or left untreated and observed at the optical microscopy for the number and morphology of attached cells. (D) The effect of H1.2 or H1.4 depletion on MCF7 and MCF10A cell proliferation was measured by following by FACS the variation of the proportion between shRNA-infected (GFP-positive) and parental cells (GFP-negative) from the 1∶1 seeding ratio over indicated days in the presence of Dox as in Figure 2B. (E) H1.2 knocked-down cells grown in the presence of Dox for 6 days were analyzed by FACS after PI staining to measure the percentage of cells in subG1phase as indicative of cell death.

In order to investigate whether the effect of H1.2 and H1.4 deletion on cell proliferation was specific to the breast cancer cell line T47D used or if it is a more extended phenotype, we introduced the inducible shRNA expression system in a different breast adenocarcinoma cell line (MCF7), a non-tumoral breast epithelial cell line (MCF10A), a cervical adenocarcinoma cell line (HeLa) and an embryonic kidney cell line (293T). Efficient H1.2 and H1.4 depletion in response to Dox treatment was obtained for all cell lines, ranging from 63 to 95% (Figure 4A and 4B). In vivo observation of cells by microscope after 6 days of Dox treatment indicated that H1.2 depletion caused a clear defect in MCF7 cell growth, but not on HeLa, 293T or MCF10A (Figure 4C). Interestingly, H1.2 depletion was more pronounced in MCF10A than in MCF-7.

In order to better describe the cell proliferation defect, H1.2 KD cells were cocultured 1∶1 with parental cells, treated with Dox and growth of the two populations was monitored by FACS along time (Figure 4D). Growth of MCF7 H1.2 KD, but not MCF7 control or MCF10A H1.2 KD, was slower than parental cells, and comparable to the results shown for T47D H1.2 KD shown in Figure 2B. Consequently, the effect of H1.2 depletion on cell proliferation was not restricted to T47D, but observed also in a different breast cancer cell line, MCF7. However, analysis of the cell cycle profile has failed to show G1 arrest. Instead a clear increase in the subG1 peak was observed in MCF7 cells (Figure 4E). It is worth noting that, unlike T47D, the MCF7 cell line is p53-positive and, consequently, prone to apoptotic death in response to adverse stimuli. This could explain the differences between the two breast cancer cell lines in response to H1.2 depletion.

On the other hand, H1.4 depletion was also obtained in the four cell lines tested, but only in MCF10A did it affect cell proliferation, as occurred in T47D (Figure 4B, 4C and 4D). Noteworthy, H1.4 depletion was stronger in HeLa cells, but no effect on cell proliferation was observed. We cannot rule out that higher depletion efficiencies (e.g. in MCF7 H1.4 KD) would produce further effects. Nonetheless, our results strongly suggest that H1.2 and H1.4 are involved in processes related to normal cell cycle progression in a cell type-dependent fashion.

### H1.2 Knock-Down Causes a Reduction in Nucleosome Spacing

**Figure 5:**
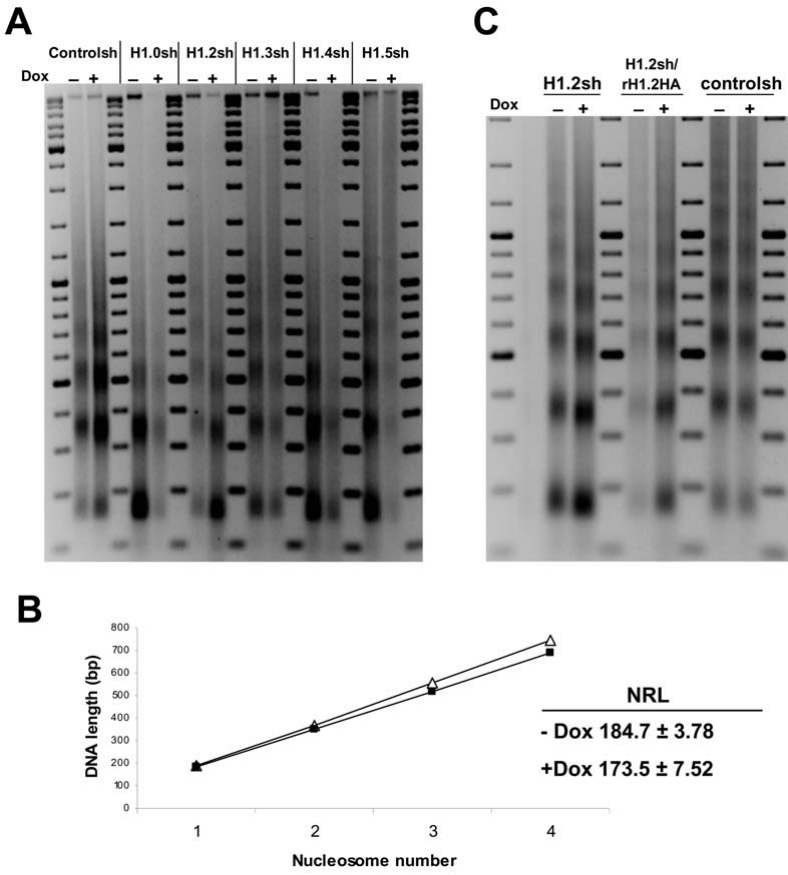
H1.2 knock-down causes a reduction in nucleosome spacing. (A) Nuclei from H1 variant knock-down cells treated or not with Dox for 6 days were treated with MNase and the profile of bulk chromatin was analyzed in gel electrophoresis to calculate the nucleosome repeat length (NRL). (B) Plot of nucleosome number versus DNA length for MNase-digested bulk chromatin of H1.2 knocked-down cells ±Dox. This plot has been used to calculate the corresponding NRL. Data from 5 independent experiments has been pooled. Average±SD is shown. Data from untreated and treated samples differed significantly in a t-Student test (95% confidence interval with a p value of 0.0001). Open triangles denote untreated, and solid square symbols correspond to cells treated for 6 days with Dox. (C) Complementation of reduced nucleosome spacing in H1.2 knocked-down cells by stable expression of shRNA-resistant H1.2 (rH1.2HA).

Reduced H1 content (down to 50%) in triple-H1 null mouse embryonic stem (ES) cells leads to reduced nucleosome spacing (nucleosome repeat length), as measured after micrococcal nuclease (MNase) digestion of nuclear bulk chromatin [12]. We investigated nucleosome spacing of chromatin from the different H1 variant T47D knock-downs by MNase digestion of isolated nuclei (Figure 5A). H1.2 depletion, but not depletion of other H1 variants, caused a striking reduction in the spacing between nucleosomes. The calculated nucleosome repeat length (NRL) was decreased from approximately 184.7 to 173.5 upon H1.2 depletion (Figure 5B, obtained from five independent experiments). This was surprising as H1.2 only represents approximately 23% of the total H1 content in T47D cells (Figure S1). As H1.2 depletion reached 90–95% and compensatory increases in the expression of other H1 variants were not detected, we estimate that total H1 content was reduced by approximately 20%. This was confirmed by Coomassie staining of H1 preparations from H1.2 KD cells treated or not with Dox (Figure S1). Stable expression of HA-tagged, shRNA-resistant H1.2 in the H1.2 KD cell line reverted the reduction in nucleosome spacing caused by endogenous H1.2 depletion (Figure 5C).

In triple-H1 null ES cells, nucleosome spacing reduction was accompanied by global changes in different histone post-translational modifications [12]. Histone modifications, such as trimethylation of K27 or trimethylation of K9 in core histone H3, remain unchanged in our H1.2 depleted cells (data not shown).

### Reduced H1.2 Content Leads Mostly to Repression of a Limited Number of Genes

**Figure 6:**
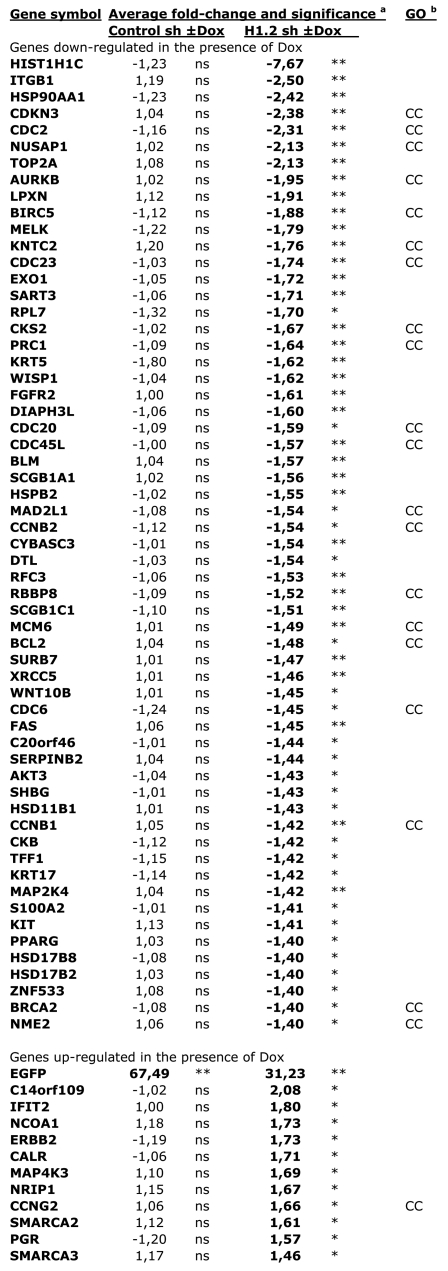
Genes with altered expression in H1.2 knock-down cells. Genes indicated have increased or decreased expression ≥1.4-fold in the presence of Dox versus in the absence of Dox (6 days)^a^. **, q≤0.05; *, 0.05≤q≤0.1; ns, non-significant. Gene onthology^b^: cell cycle-related genes are identified with CC. The nomenclature of genes is shown in Table S5.

H1.2 depletion alters global chromatin structure as seen by the reduction in nucleosome spacing. This could have some effect on nuclear processes such as DNA replication or gene expression and, ultimately, cell proliferation. We studied the effect of H1.2 depletion on global gene expression. For this, we used a custom-made breast cancer microarray platform containing 826 cDNA clones (see Text S1) to compare H1.2 KD cell line treated for 6 days with Dox with untreated cells and with control cells ±Dox. In order to avoid comparing cells arrested in the cell cycle (H1.2 KD +Dox) with cells normally cycling (H1.2 KD −Dox), all cells were equally arrested in G1 by serum deprivation over the last two days of ±Dox treatment prior to RNA extraction (Figure 2E). Depletion of H1.2 caused an alteration in the expression of a limited number of genes. Most of altered genes were repressed in the absence of the histone: 11 genes were up-regulated and 59 genes were down-regulated with a fold-change higher than 1.4 (q≤0.1), with the H1.2-encoding gene (hist1h1c) being the strongest down-regulated cDNA due to the shRNA specific action (Figure 6). These changes were not observed in control cells, discarding an effect of Dox on gene expression. This indicates that H1.2, rather than being a general repressor, is acting direct or indirectly as positive regulator of gene expression. Among the repressed genes, many of them are involved in cell cycle control. Gene ontology identified 20 out of the 59 repressed genes as being related to cell cycle, and 10 to cell division. This proportion is higher than would be expected, considering the abundance of these gene categories in the customized array. Alteration in the expression of one or a subset of cell cycle genes could cause the G1 arrest.

### Depletion of the Different H1 Variants Leads to Specific Changes in Global Gene Expression

**Figure 7:**
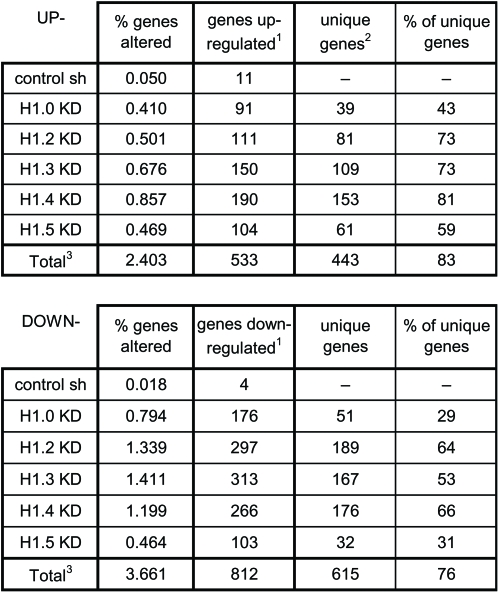
Summary of analysis of gene expression in H1 variant KD cells with genome-wide microarrays. ^1^ Number of genes with increased or decreased expression ≥1.4 fold (q≤0.1) in the presence of Dox versus in the absence of Dox (6 days). ^2^ Number of genes altered by depletion of a particular isoform only. ^3^ Total refers to the genes altered by depletion of some of the H1 isoforms. Genes affected by more than one isoform are counted only once.

The analysis of the consequences of H1 depletion on global gene expression was extended to the other H1 variants using genome-wide Illumina microarrays containing more than 22,000 probes (see Text S1). All KD cell lines were treated or not with Dox for 6 days, in duplicate, and arrested in G1 by 48 h of serum-starvation prior to RNA extraction. A summary of the number of genes up- or down-regulated with a fold-change ≥1.4 (false discovery rate q≤0.1) in each H1 variant KD cell line upon Dox treatment in comparison to untreated cells is presented in Figure 7 and Tables S2 and S3.

The proportion of altered genes, up- or down-regulated, ranged between 1 and 2-%, depending on the H1 variant under study. Globally, approximately 6% of the genes are altered by depletion of some of the variants. H1 depletion caused 1.5 times more down- than up-regulation of genes. In particular, the ratio down- to up-regulated ranged between 1 for H1.5 and 2.7 for H1.2. As reported above, H1.2 depletion mostly leads to gene down-regulation.

A total of 533 genes were up-regulated by depletion of some H1 variant. Of these, 443 (83%) were unique to a single variant. Seventy genes were up-regulated by two H1 variants, and 20 by three or four variants. The number of genes down-regulated by depletion of some variant was 812. Of these, 615 (76%) were uniquely regulated, 111 genes were regulated by two variants, and 86 by more than two variants. Interestingly, H1.2, H1.3 and H1.4 were the variants that produced a higher proportion of uniquely-regulated genes, indicating that these are the variants with a more specific role in gene expression control. On the other hand, the majority of genes down-regulated in H1.0 and H1.5 KD were also affected by depletion of other H1 variants.

In conclusion, depletion of the different H1 variants caused an alteration in the expression of a limited number of genes, different in each H1 variant KD. Most of the genes are affected by a single H1 variant, supporting the theory that H1 isoforms play specific roles in gene expression. Nonetheless, a proportion of genes are altered by more than one H1 variant, suggesting that redundant roles for H1 variants may also exist.

### Expression of Cell Cycle-Related Genes Is Altered in H1.2-Depleted Cells

**Figure 8:**
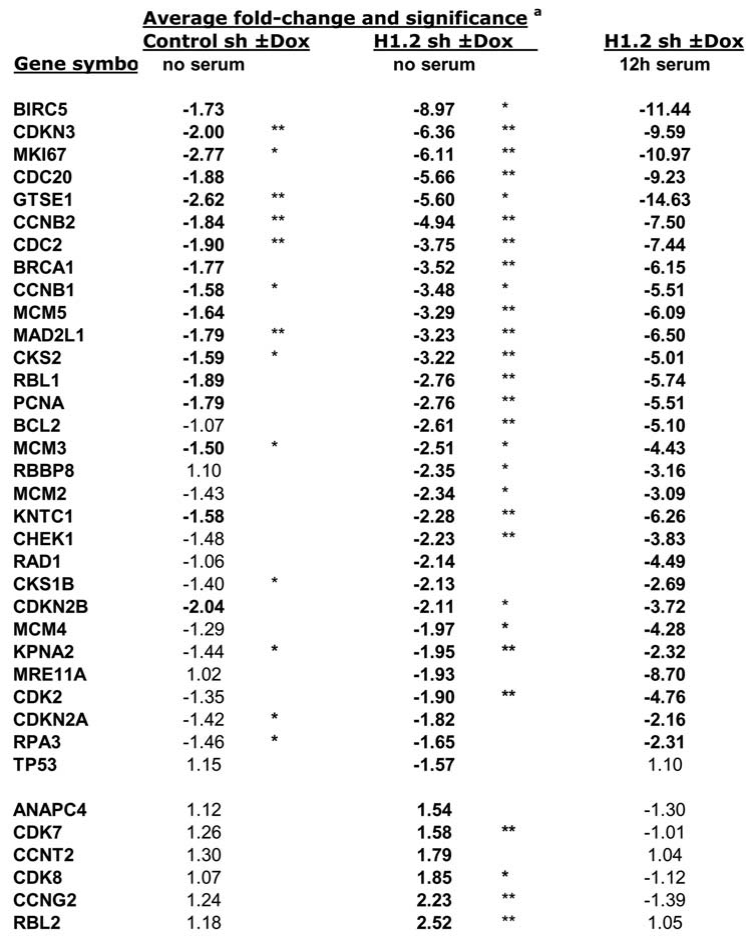
Cell cycle-related genes with altered expression in H1.2 knock-down cells. Genes indicated have increased or decreased expression ≥1.5-fold in the presence of Dox versus in the absence of Dox (6 days)^a^. **, q≤0.05; *, 0.05≤q≤0.1. The nomenclature of genes is shown in Table S6.

To further explore the relationship between H1.2 depletion and the cell cycle gene expression profile, we tested expression of 84 cell cycle-related genes by RT-real time PCR using the SuperArray (Bioscience Corporation) platform (Figure 8). Fourteen genes were significantly repressed (more than 2-fold) when H1.2 was depleted after Dox addition for 6 days in serum-starved cells (q≤0.5). This number was increased to 24 repressed genes when considering ≥1.5-fold and q≤0.1, and included 10 genes already identified in the customized microarray experiments: *BIRC5*, *CDKN3*, *CDC20*, *CCNB2*, *CDC2*, *CCNB1*, *MAD2L1*, *CKS2*, *BCL2* and *RBBP8*. Among the newly identified genes were *CDK2*, *PCNA*, *MCM2*, *MCM3*, *MCM5*, *CDKN2A* (p16^INK4A^) and *CDKN2B* (p15^INK4B^). Only two genes were activated ≥2-fold: *RBL2* and *CCNG2*. In the same experiment, cells kept ±Dox (6 days) and serum-starved (last two days) were further incubated with 10% serum for an additional 12 hours period and RNA was extracted. Differences in expression of the repressed genes were even higher, confirming that expression of this subset of genes was affected as a consequence of H1.2 depletion (Figure 8).

**Figure 9:**
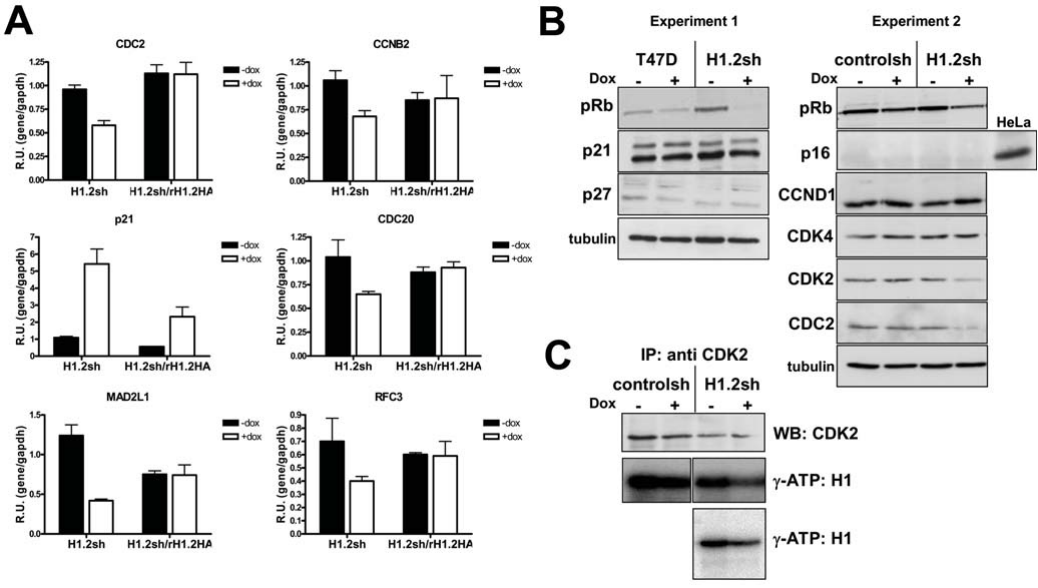
Cell cycle gene alterations in H1.2 knock-down cells. (A) Complementation of altered expression of cell cycle-related genes in H1.2 knocked-down cells by stable expression of recombinant HA-tagged, shRNA-resistant H1.2. Expression of the cell cycle genes indicated was measured by RT-qPCR with specific oligonucelotides in parental H1.2 KD and H1.2sh/rH1.2HA cells treated or not with Dox for 6 days. GAPDH expression was measured for normalization. Data is expressed as relative units specific gene/GAPDH. The values represent the mean and SD of a representative experiment performed in triplicate. (B) Changes in the accumulation or phosphorylation of cell cycle-related proteins upon inhibition of H1.2. Expression of the indicated proteins was measured by Western blot with specific antibodies in cells treated or not with Dox for 6 days. H1.2 knocked-down cells were compared to parental T47D or control cells. Tubulin antibody was used as loading control. (C) CDK2 activity in H1.2 depleted cells. CDK2 was immunoprecipitated with the specific antibody from cellular extracts of Dox-treated or untreated cells (H1.2 KD or control) and incubated with histone H1 and γ-ATP. Products were resolved in SDS-PAGE and detected by autoradiography. Immunoprecipitated material was measured by immunoblotting with the anti CDK2 antibody.

Expression of some of the identified genes was further analyzed by conventional RT-qPCR with specific oligonucleotides. Interestingly, we detected induction of the cell cycle inhibitor *CDKN1A* (p21^Cip1^), which had not been observed using the previous methods. Some of the genes were repressed (*CDC2*, *CDKN3*, *RFC3*) or activated (*CDKN1A*) as early as 2 days after Dox addition. Other genes were not repressed until day 6 (*CDC20*) (Figure S4). Next, we tested whether inhibition of genes in H1.2 depleted cells could be reverted by stable reintroduction of recombinant H1.2 (Figure 9A). Inhibition of *CDC2*, *CCNB2*, *CDC20*, *MAD2L1* and *RFC3* was reverted by restoring H1.2 levels. Induction of *CDKN1A* (p21^Cip1^) was only partially reverted by reintroduction of H1.2.

Expression of cell cycle-related genes may vary during cycle progression. We investigated the effect of H1.2 depletion on the expression of these genes over time after release from a serum-starvation induced G1-arrest block. In H1.2-depleted cells (Dox-treated), the response of the genes to serum was, in most of the cases, delayed and reduced (Figure S4).

In order to test whether repression of this cell cycle gene subset was specific to H1.2 depletion, we tested gene expression in control, H1.0, H1.2 and H1.4 knock-down cells ±Dox, in parallel, with the SuperArray platform (Table S4). Twenty-seven genes were repressed (≥1.5-fold) in comparison to control cells ±Dox when H1.4 was depleted, with approximately 50% coincidence with genes repressed upon H1.2 inhibition. This included *CDKN3*, *MAD2L1* and *CCNB2*. Only one gene was slightly activated. On the other hand, H1.0 depletion caused activation of approximately 20 genes (≥1.5-fold) and repression of only 2 genes. Our results indicate that depletion of different H1 variants affect expression of cell cycle-related genes diversely.

### Cell Cycle Protein Profile in H1.2-Depleted Cells

In order to better characterize the G1 arrest caused by H1.2 depletion, we analyzed accumulation or phosphorylation of several cell cycle regulators in ±Dox-treated H1.2 KD and control cells by Western blot with specific antibodies (Figure 9B). Phosphoryation of retinoblastoma protein (Rb) is a key gatekeeper of G1 to S transition through the restriction point of the cell cycle [40],[41]. Rb phosphorylation was highly reduced in Dox-treated H1.2 KD cells, but not in control cells, as shown in two different experiments. Higher phoRb in H1.2 KD (−Dox) compared to parental T47D observed in experiment 1 was not reproducible. Rb phosphorylation is required for transcription of S-phase genes, e.g. *CDC2*, *PCNA* and cyclins E/A. Some of them are consistently down-regulated in H1.2 KD, such as *CDC2* and *PCNA*. Accordingly, the level of CDC2 protein was reduced in H1.2 KD cells +Dox (Figure 9B).

Rb is consecutively phosphorylated in the G1 phase by the CDK4/6-Cyclin D1 and CDK2-Cyclin E pairs. We did not detect changes in the accumulation of Cyclin D1 or CDK4 proteins (Figure 9B). Instead, CDK2 levels were reduced in H1.2 KD, as well as the CDK2 -associated kinase activity immunoprecipitated from these cells (Figure 9B and 9C). *CDK2* gene expression was also reduced upon H1.2 depletion (Figure 8 and Table S4). As with CDC2, CDK2 down-regulation in H1.2 KD cells could also help to explain the G1 arrest.

In the DNA damage response, p53-dependent induction of expression of the CDK inhibitor p21^Cip1^ is involved in the defect in Rb phosphorylation [42]. In our experiment, the levels of the CDK inhibitors p21^Cip1^ and p27^Kip1^ did not change (Figure 9B), although *CDKN1A* gene expression was increased in H1.2-depleted cells (Figure 9A). Considering that T47D cells are p53-defective, we cannot rule out that a different player affected ultimately by H1.2 depletion, such as inhibitors of the INK4 family, is impacting on Rb phosphorylation. One of these inhibitors, p16^INK4A^ was not detected in our cells (Figure 9B). We can conclude that G1 arrest in H1.2 KD cells is caused by deregulation of cell cycle factors upstream the phosphorylation of Rb, but its exact causes still remain elusive to us as does, more importantly, the way in which H1.2 governs regulation of these factors.

## Discussion

Histone H1 is a structural component of chromatin related to compaction and inaccessibility to transcription factors or RNA polymerase. Nonetheless, it has been suggested that histone H1 plays a more dynamic and gene-specific role, participating in activation or repression of gene expression. Moreover, it is not fully understood to what extent H1 subtypes are functionally redundant or exert specific functions. Previous reports indicate that distinct H1 variants may have different properties and implications for gene expression [8],[18],[43],[44]. Our analysis by means of inducible RNA interference allows for investigation of the phenotypic and gene expression effects of variant-specific H1 depletion on a human breast cancer cell line, avoiding compensatory up-regulation of other H1 subtypes or epigenetic adaptation to chromatin structure changes. We have confirmed that depletion of H1 subtypes alters expression of different subsets of genes. Particularly, we found that depletion of H1.2 leads mainly to down-regulation of genes, supporting a positive role for this H1 variant in gene expression, rather than a general repressive role.

### H1 Depletion Impacts on Cell Cycle Progression in a Cell Type and H1 Variant-Specific Manner

We have investigated the consequences of sudden depletion of a particular H1 subtype. We found that H1.2 and H1.4 have an essential role in normal growth of a human breast cancer cell line in culture. On the other hand, 70–90% depletion of H1.0, H1.3 or H1.5 subtypes was compatible with normal cell growth. We cannot discard that further depletion of these isoforms could have additional consequences. To our knowledge, no previously reported features of H1 subtypes anticipated these results. H1.0 is highly abundant in terminally differentiated cells, while somatic H1 variants (H1.1-H1.5) are present in dividing cells with differing abundance depending on cell type. Interestingly, no human cell line lacking expression of H1.2 or H1.4 has been found to date, supporting the important role of these two isoforms [45],[46]. These reports show that all cell types tested express H1.2 and H1.4 at a high level, each accounting for at least 20% of the cellular H1 content. In contrast, cell types can be found where some of the other H1 subtypes are not detectable or at very low levels (≤5% of total H1). This is in agreement with our estimations of the H1 content in the five cell lines used in this report. Analysis of the H1 variants pattern in T47D cells combining gel electrophoresis and immunoblotting (Figure S1 and data not shown) indicated that the relative contribution of subtypes to the total H1 content was approximately as follows: 9% for H1.0, 23% for H1.2, 13% for H1.3, 24% for H1.4 and 31% for H1.5.On the other hand, the amount of H1.5 was low in HeLa and 293T, H1.3 was undetectable in HeLa, and H1.0 almost inexistent in MCF10A (Figure 4).

Histone H1.4 depletion had a strong impact on cell survival of T47D cells. This cell line is functionally defective in p53 and caspase-3, and consequently these cells do not undergo apoptosis in response to stress or damage. Indeed, we have been unable to detect signs of apoptotic death upon H1.4 depletion. Six days after induction of H1.4 KD, few cells remained attached to the culture flask, and these cells presented a necrotic phenotype. When the cell cycle profile was analyzed, very few cycling cells were detected and FACS analysis identified the presence of cells in the subG1 window. Gene expression profiles showed the alteration in expression of a considerable number of cell cycle related genes. Although our data suggests that H1.4 is essential for T47D cell growth, at this point it cannot be discarded a more complicated explanation as a consequence of indirect effects of H1.4 depletion, such as the resulting increased expression of H1.0.

Depletion of H1.2 in T47D cells caused G1 arrest and, consequently, slow proliferation. This effect is specific to H1.2 depletion, as it did not occur in KD cells for the other variants and it was complemented solely by the reintroduction of recombinant, shRNA-resistant H1.2.

The effects of H1.2 or H1.4 depletion are not unique to the T47D cell line, but are also observed in MCF7 and MCF10A cells, respectively. Noteworthy, H1.2 depletion did not cause G1 arrest in MCF7 but rather an apoptotic phenotype. Differences in genetic background between cell lines may explain different outcomes for the same stress. For instance, unlike T47D, MCF7 is p53-positive and prone to apoptosis. Cell lines from other origins did not exhibit a cell cycle phenotype in response to H1.2 KD. This is perplexing as H1.2 accounts for almost 50% of the total H1 content in HeLa (Figure 4A and [46]), and is also highly abundant in 293T. This suggests that the effect of H1 depletion is cell type specific and unlinked to the relative amount of each variant in the cell, nor correlates with the efficiency of depletion achieved by RNA interference.

Tumor cells undergo uncontrolled proliferation, arising from the ability to bypass the quiescent state characteristic of most normal cells from adult tissues [41]. H1.2 depletion blocks the proliferative potential of breast cancer cells and therefore unraveling the underlying mechanism could provide insights into how normal cells become tumorigenic.

### Effect of H1.2 Depletion on Nucleosome Spacing and Chromatin Organization

Triple null H1-2-H1.3-h1.5 mouse embryonic stem (ES) cells present a 15 base pair reduction in the bulk chromatin NRL [12]. H1.2 depletion of breast cancer cells altered chromatin organization, measured as nucleosome spacing after controlled MNase digestion of nuclei. NRL was decreased by about 11 bp upon H1.2 depletion, and the effect was specific to this subtype and complemented by recombinant H1.2 expression. This was unexpected as a reduction of NRL has been reported only after a significant (50% or higher) decrease in H1 content [12]. We estimated that H1.2 accounts for only about 20–25% of the total H1 content in T47D cells and depletion by shRNA was not complete. In our experiments, depletion of other H1 subtypes accounting for a similar or higher percentage of total H1 (H1.4 and H1.5) had no effect on chromatin structure, indicating that H1.2 may play a specific role in chromatin organization or nucleosome spacing.

Triple KO mouse ES cells proliferate at the same rate as WT ES cells and also exhibit a decrease in the levels of some core histone modifications as well as in CpG methylation within the regulatory regions of certain genes [12]. So far, we have not detected changes in global histone modifications nor in DNA methylation upon H1.2 depletion (data not shown). Using a microarray platform to measure methylation of CpG loci in 807 genes (including cell cycle control, apoptosis, chromosome X-linked, and imprinted genes), we have not observed significant changes in H1.2 KD cells treated or not with Dox for 6 days (M. Sancho, A. Fernández, M. Beato, M. Esteller, A. Jordan; unpublished results). Epigenetic changes to compensate for H1 depletion in ES cells may require longer times and may allow cell survival under selective pressure, whereas rapid induction of H1.2 shRNA does not allow for compensation effects to appear before cell progression is compromised.

We have not yet been able to establish a causative relation between the reduced nucleosome spacing and the cell cycle arrest in G1 phase observed upon H1.2 depletion in T47D. Alterations of chromatin organization could affect progression of the replication fork, or could expose to damage regions of DNA leading to a damage response. It has been recently reported that H1-depleted cells (triple KO ES cells) are hyper resistant to DNA damage and present hypersensitive checkpoints (G2/M arrest induced at lower doses), explained by increased signaling generated at each DNA break [47]. If so, upon H1.2 depletion we would expect S or G2/M arrest in response to damaging agents such as HU or MMS, respectively. But we have clearly shown that G1-arrest is dominant with regard to arrest caused by such compounds.

H1 depletion could also have an effect on the formation and stability of mitotic chromosomes. In the absence of H1, chromosomes assembled and replicated in Xenopus egg extracts failed to compact properly, leading to segregation anomalies [48]. In triple KO mouse ES cells, no abnormalities in mitosis and normal division time were detected [12]. Nonetheless, the decrease in H1 leads to a general increase in telomere length [47]. It remains to be explored if selective H1.2 depletion leads to chromosomal abnormalities.

### Changes in Expression of Cell Cycle Genes

Chromatin missorganization could also cause changes in the expression of sensitive promoters that may include genes relevant for cell proliferation. Alternatively, H1.2 may be a specific regulator of some particular promoters. It has been reported before that H1.5 interacts with transcription factor MSX1 and both are recruited to specific promoters to control myogenesis [19]. On the other hand, H1.2 has been found to be part of a complex that represses p53-mediated transcription in HeLa cells [24]. With this idea in mind, we have explored what changes in gene expression profiles occur upon depletion of different H1 subtypes. Less than 10% of the genes present in a customized microarray (and almost 2% on a genome-wide array) were altered in its expression upon H1.2 knock-down and most of them were repressed. This behavior has been reported before [14]–[16] and is indicative of H1 acting as an active regulator of gene expression, not solely a repressor due to its chromatin compaction properties. Repressed genes included a relevant proportion of cell cycle-related genes. We reject the possibility that this is due to different proportion of cells in the different cell cycle phases upon exposure to the shRNA, as all cell populations were equivalently arrested in G1 due to serum-starvation for the gene expression experiments. Rather we found an effect of H1.2 deprivation on a subset of cell cycle related genes, some of which are in line with the G1 arrest phenotype and may be the link to the cell proliferation defect.

Replicative or premature, stress-induced senescence is characterized by maintenance of cells in a growth-arrest state (comparable to G1 arrest), by forming heterochromatin at proliferation-promoting gene loci (E2F target genes). It has recently been suggested that alteration in histone H1 metabolism may be involved in a cellular senescence-inducing mechanism [49]. Senescent fibroblasts show reduced levels of chromatin-bound endogenous H1. That report did not clarify whether histone H1 loss plays a causative role in senescence induction or is only an effect of the process, as RNA interference of H1 was unsuccessful. Our data suggests that specific H1 subtype loss from chromatin may cause by itself this phenotype in T47D cells, indicating that H1 loss may be a messenger between senescence-inducing signals and the molecular response leading to growth arrest.

G1 progression and entry into S phase depends on the activity of two cell cycle kinase-complexes, CDK4/6-cyclin D and CDK2-cyclin E, that work in concert to relieve inhibition of a dynamic transcription complex that contains retinoblastoma (Rb) protein and E2F. Phosphorylation of Rb by CDK4/6 and CDK2 dissociates the Rb-HDAC repressor complex, permitting transcription of key S-phase-promoting genes, including some required for DNA replication [40]. Downstream genes controlled by E2F, such as *CDC2* and *PCNA* are repressed in the absence of H1.2. CDKs activity is regulated by members of the INK4 (p15, p16, p18, p19) or KIP/CIP (p27, p21) families of cyclin dependent kinase inhibitors induced by different agents or growth conditions promoting cell cycle arrest, differentiation or apoptosis. In H1.2 knock-down cells (+Dox), Rb remains unphosphorylated when compared with −Dox cells, indicating that the cause of G1 arrest is upstream Rb phosphorylation by CDKs. p16^INK4A^, p27^Kip1^ and p53-inducible p21^Cip1^ could be candidates to explain low Rb phosphorylation, but we have not appreciated any difference in the level of either inhibitor in H1.2 KD cells, despite the fact that the *CDKN1A* (p21^Cip1^) gene is induced upon H1.2 depletion. Involvement of other inhibitors of the INK4 family needs to be tested. Cyclin D1 and CDK4 levels also remain unaltered. On the contrary, CDK2 and CDC2 levels are reduced in H1.2 KD cells. The CDC2 (CDK1)-Cyclin E pair has been involved recently in G1/S transition and could substitute CDK2, and so it could also account for Rb phosphorylation [50],[51]. Reduced levels of these two proteins may account for cell cycle arrest by itself, or in combination with additional yet unrecognized factors. Among the repressed genes in H1.2 KD cells, there are genes involved in initiation of replication such as those encoding for CDC6, CDC45L, PCNA and several MCM proteins. Down-regulation of these factors could prevent entry into S phase and, consequently, cells would remain in G1. Another set of repressed genes encode for proteins involved in G2/M transition, such as CDC2, and mitosis progression, such as MAD2L1, CDC20 and CDC23, cyclin B, aurora kinase B. Their repression does not explain the G1 arrest, but may be part of a gene network co-regulated by H1.2 depletion.

It is difficult to unequivocally assign a gene or group of genes as direct targets of the H1.2 depletion or as responsible of the G1 arrest phenotype. The first would require investigation into the participation of this particular histone subtype in the promoter activation process of candidate genes by chromatin immunoprecipitation, but H1 subtype specific antibodies generated have been useless to date for this application. The second would require testing the effect of interfering with each cell cycle-related candidate gene. Probably, simultaneous, incomplete silencing of several genes would be needed to recapitulate the G1 arrest observed here [52]. Our data and data published elsewhere are indicative that depletion of cell cycle regulatory proteins such as CDC2, CDK2, CDC20, MCMs or PCNA is deleterious for cell cycle progression [53]–[55].

### H1 Variants Play Specific and Redundant Roles in Gene Expression Regulation

Global gene expression profiling combining inducible H1 variant depletion and genome-wide microarrays has allowed us to define the subsets of genes regulated directly or indirectly by each variant. The overall number of regulated genes and the proportion of genes up- or down-regulated differ between H1 isoforms. Depletion of the different H1 variants caused an alteration in the expression of a limited number of genes, different in each H1 variant KD. Most of the genes (79%) are affected by a single H1 variant, supporting the theory that H1 isoforms play specific roles in gene expression. Nonetheless, a proportion of genes are altered by more than one H1 variant, suggesting that redundant roles for H1 variants in gene expression regulation may also exist. 13% of genes were altered in two variant KDs, and only 1% of genes were affected (down-regulated) in each of the five KDs. This is in agreement with a recent report showing that overexpression of mouse H1^0^ and H1c (human H1.0 and H1.2 orthologs) altered a limited number of variant-specific genes, as well as a smaller number of commonly regulated genes [16]. In that case, too, genes were either up- or down-regulated upon H1 overproduction, with a stronger predominance of up-regulated genes in the H1c-overexpressing cells and a major number of down-regulated genes in the H1^0^ cell line. Similarly, we have obtained a higher ratio down- to up-regulated genes in the H1.2 KD (ratio DW:UP = 2.7) than in the H1.0 KD (ratio = 1.9). This supports the theory that H1.0 is a stronger repressor of transcription and H1.2 may play a role as transcription activator or facilitator. Nonetheless, following with this argument, H1.4 and H1.5 would be even more repressive (ratio = 1.4 and 1, respectively). This, actually, fits with previous description of histone H1 variant distribution and compaction capability. It has been proposed that mouse H1.0, H1.4 and H1.5 promote formation of compact chromatin and are enriched in more compact chromatin, while H1.2 condenses chromatin less effectively and is enriched in less condensed chromatin regions [8],[56].

Our analysis has also shown that H1.3, and especially H1.2 and H1.4, are the most specific variants in controlling gene expression, with a higher number of uniquely regulated genes. This might be related to the essentiality of H1.2 and H1.4 in breast cancer cells reported here, and with the fact that no cell types lacking any of the two variants have been found to date. These two isoforms may play more specific roles, while the other variants may be more redundant. This is still speculation and to proof it would need to cross the different H1 variant knock-downs with all the vectors for ectopic expression of HA-tagged recombinant isoforms.

Considering that histone H1 is a global chromatin component, our and previous reports studying global gene expression changes in response to H1 depletion [12]–[15], show that H1 affects expression of a surprisingly reduced number of genes. Here, approximately 6% of the genes present in the 22K-gene microarray show altered expression (>1.4-fold) upon inhibition of some of the five H1 variants tested. Nevertheless, not all the genes present in the microarray platform are expressed in this breast cancer cell line growing under the conditions used, i.e. G1 arrest caused by serum starvation. Only about 36% of the genes were expressed above background (data not shown). This increases the proportion of genes being affected by depletion of some H1 variant in up to 16% of expressed genes (6.6% up- and 10% down-regulated by H1 depletion). This is already a considerable number of affected genes, larger than the 0.56% of expressed genes affected (>2-fold) in mouse triple H1 knock-out embryonic fibroblasts published elsewhere [12]. If we also consider a >2-fold variation in gene expression for the analysis of data, 2% of expressed genes are still affected upon depletion of some H1 variant. The striking difference between the two studies —with all due caution with regard to differences in the data analysis procedure applied— might be due to the inducible nature of the knock-down strategy used here. Along a small number of cell divisions, cells are encountered with a reduced level of a particular isoform. In the triple mutant, cells have been selected for survival and have adapted to the new situation, allowing for the resetting of a hypothetical basal gene expression profile required for the maintenance and progression of cells.

In conclusion, our approach of inducible depletion of individual H1 variants using RNA interference technology has revealed that H1 subtypes are not fully redundant but may have differentiated functions in some of their nuclear roles, including nucleosome spacing and gene expression control, impacting on important cellular events such as proliferation.

## Supporting Information

Figure S1 Characterization of human histone H1 variant-specific antibodies and analysis of the H1 variants pattern in T47D cells.
(0.15 MB PDF)

Figure S2 Rescue of the deleterious effect of H1.4 shRNA by transient expression of recombinant shRNA-resistant H1.4.
(0.14 MB PDF)

Figure S3 Incorporation of recombinant HA-tagged H1 isoforms into chromatin.
(0.05 MB PDF)

Figure S4 Cell cycle gene alterations in H1.2 knock-down cells.
(0.03 MB PDF)

Table S1 Cell cycle profile after PI staining of H1 variant KD cell lines grown in the presence or absence of Dox.
(0.01 MB PDF)

Table S2 Summary of gene expression altered upon H1 variant depletion.
(0.01 MB PDF)

Table S3 Number of genes regulated uniquely or coincidently between different isoforms.
(0.01 MB PDF)

Table S4 Effect of H1 variant inhibition on the expression of 84 genes key to cell cycle regulation.
(0.02 MB PDF)

Table S5 Nomenclature of genes shown in Figure 6.
(0.02 MB PDF)

Table S6 Nomenclature of genes included in the RT Profiler PCR Array.
(0.02 MB PDF)

Text S1 Suppplementary figure legends and Materials & Methods.
(0.12 MB PDF)
