# Supplementary figures and images for: Depletion of Human Histone H1 Variants Uncovers Specific Roles in Gene Expression and Cell Growth
Source: PLoS Genet. 2008 Oct 17;4(10):e1000227. doi: 10.1371/journal.pgen.1000227 (PMC2563032; doi:10.1371/journal.pgen.1000227)

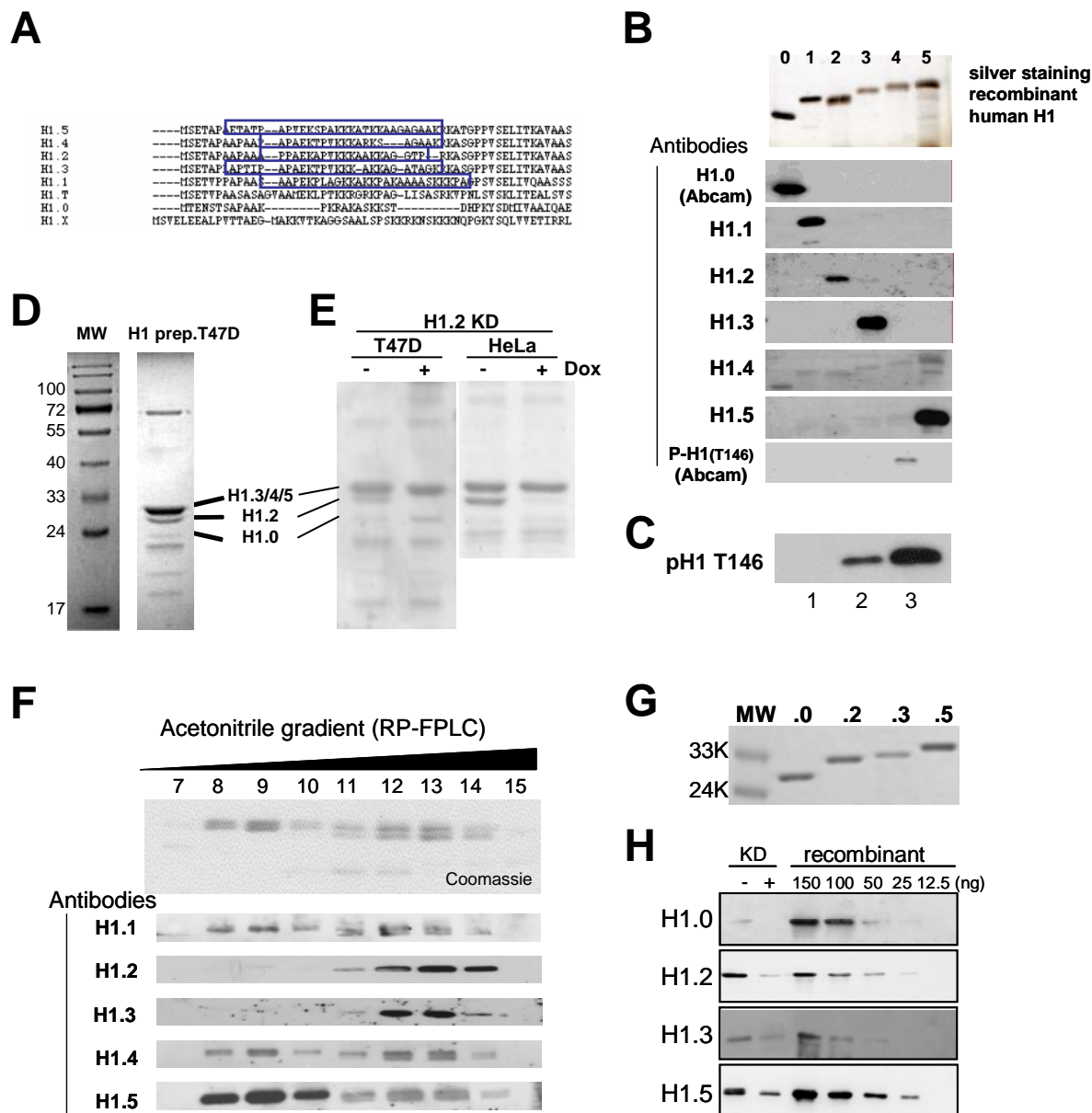

Figure S1

Supplement: Figure S1 — Characterization of human histone H1 variant-specific antibodies and analysis of the H1 variants pattern in T47D cells. (0.15 MB PDF) [file pgen.1000227.s001.pdf]

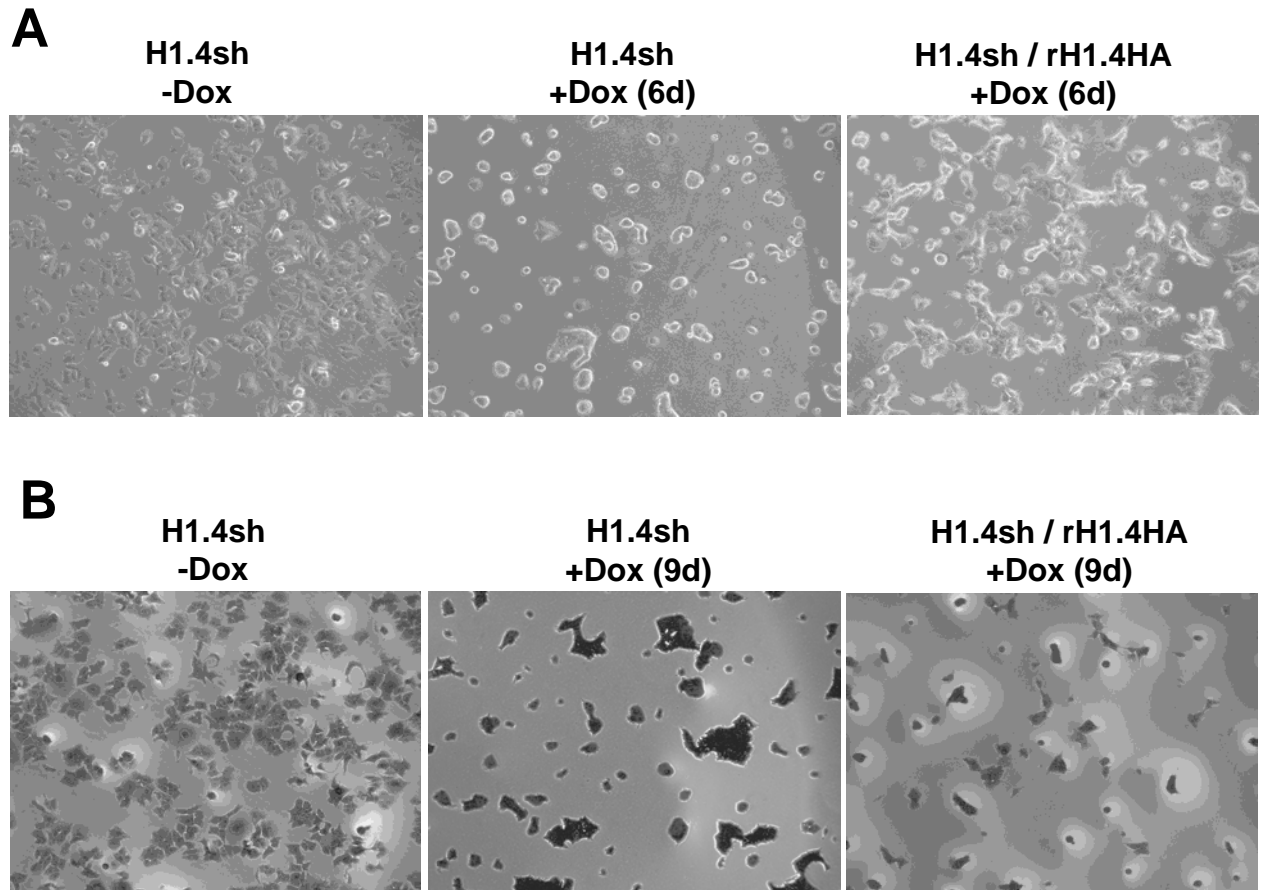

**Figure S2**

Supplement: Figure S2 — Rescue of the deleterious effect of H1.4 shRNA by transient expression of recombinant shRNA-resistant H1.4. (0.14 MB PDF) [file pgen.1000227.s002.pdf]

**A**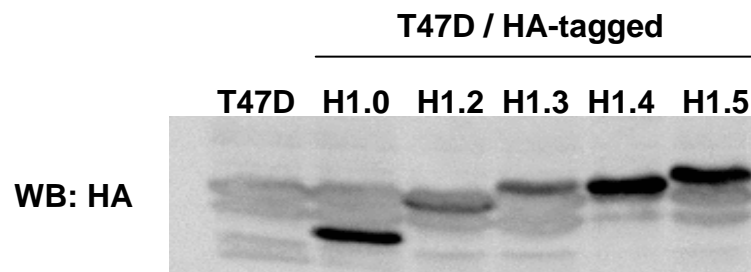**B**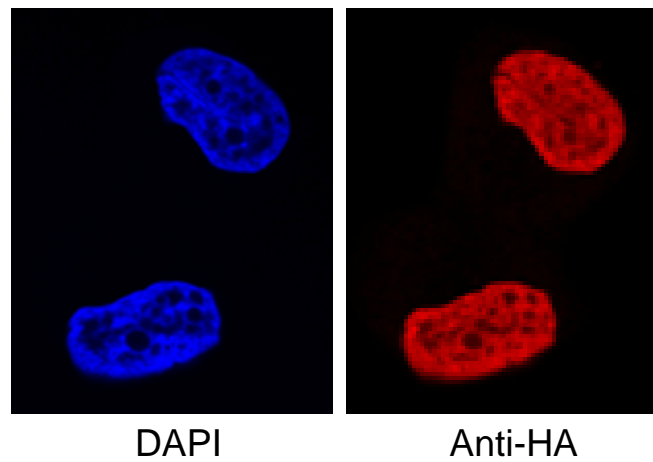**C**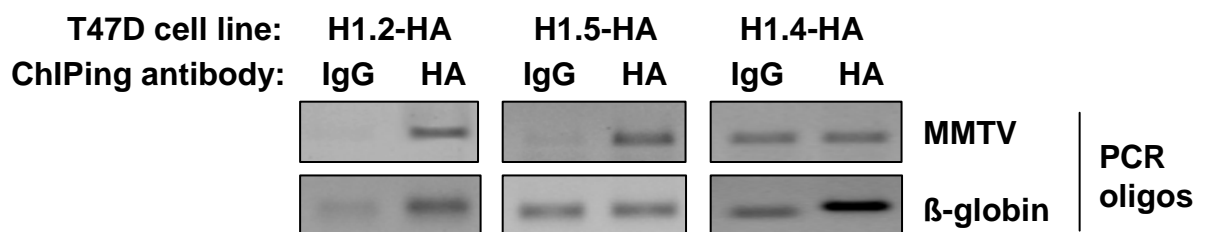**Figure S3**

Supplement: Figure S3 — Incorporation of recombinant HA-tagged H1 isoforms into chromatin. (0.05 MB PDF) [file pgen.1000227.s003.pdf]
