# Supplementary material for: Depletion of Human Histone H1 Variants Uncovers Specific Roles in Gene Expression and Cell Growth
Source: PLoS Genet. 2008 Oct 17;4(10):e1000227. doi: 10.1371/journal.pgen.1000227 (PMC2563032; doi:10.1371/journal.pgen.1000227)
Supplement: Figure S4 — Cell cycle gene alterations in H1.2 knock-down cells. (0.03 MB PDF) [file pgen.1000227.s004.pdf]

Table S1. Cell cycle profile after PI staining of H1 variant KD cell lines grown in the presence or absence of Dox. See legends of Figures 2C and 2E for a full description of the experimental procedure.

Data for Fig. 2C

	Dox	G1-phase	S-phase	G2-phase
controlsh	-	68.88 ± 1.83	9.10 ± 0.80	20.62 ± 1.14
	+	67.45 ± 3.00	8.43 ± 1.86	22.05 ± 4.05
H1.0sh	-	70.89 ± 1.46	9.02 ± 0.61	18.76 ± 0.61
	+	70.15 ± 2.76	9.65 ± 1.42	18.69 ± 1.35
H1.2sh	-	68.56 ± 0.93	10.87 ± 0.04	19.77 ± 0.93
	+	82.36 ± 2.83	4.17 ± 0.39	12.63 ± 2.66
H1.3sh	-	69.67 ± 1.99	8.92 ± 1.57	19.96 ± 0.17
	+	69.00 ± 1.08	8.00 ± 0.28	21.45 ± 1.16
H1.4sh	-	72.63 ± 0.13	9.00 ± 0.03	17.34 ± 0.76
	+	75.05 ± 3.92	2.30 ± 0.37	21.73 ± 3.81
H1.5sh	-	69.16 ± 1.29	11.19 ± 2.47	18.04 ± 1.92
	+	71.10 ± 2.41	5.78 ± 1.62	21.82 ± 2.07

Data for Fig. 2E

H1.2sh KD

FBS (h)	Dox	G1-phase	S-phase	G2-phase
0	-	88.07 ± 1.46	2.53 ± 0.53	11.23 ± 1.52
24	-	42.58 ± 0.82	32.20 ± 0.13	23.40 ± 1.34
30	-	39.67 ± 4.24	18.59 ± 0.41	39.85 ± 4.70
48	-	66.37 ± 3.49	11.66 ± 0.57	20.64 ± 2.35
0	+	85.34 ± 1,54	2.58 ± 0.40	11.87 ± 1.22
24	+	85.40 ± 0.41	5.67 ± 0.87	7.93 ± 0.64
30	+	71.37 ± 4.40	14.35 ± 1.77	12.57 ± 2.31
48	+	72.11 ± 2.09	5.65 ± 0.28	20.85 ± 1.00
