# Supplementary material for: Depletion of Human Histone H1 Variants Uncovers Specific Roles in Gene Expression and Cell Growth
Source: PLoS Genet. 2008 Oct 17;4(10):e1000227. doi: 10.1371/journal.pgen.1000227 (PMC2563032; doi:10.1371/journal.pgen.1000227)
Supplement: Table S2 — Summary of gene expression altered upon H1 variant depletion. (0.01 MB PDF) [file pgen.1000227.s006.pdf]

Table S2. Summary of gene expression altered upon H1 varinat depletion.

TOTAL	% genes altered	ratio DW:UP
control sh	0.068	0.364
H1.0 KD	1.204	1.934
H1.2 KD	1.840	2.676
H1.3 KD	2.088	2.087
H1.4 KD	2.056	1.400
H1.5 KD	0.933	0.990
Total	6.065	1.523
