# Supplementary material for: Depletion of Human Histone H1 Variants Uncovers Specific Roles in Gene Expression and Cell Growth
Source: PLoS Genet. 2008 Oct 17;4(10):e1000227. doi: 10.1371/journal.pgen.1000227 (PMC2563032; doi:10.1371/journal.pgen.1000227)
Supplement: Table S3 — Number of genes regulated uniquely or coincidently between different isoforms. (0.01 MB PDF) [file pgen.1000227.s007.pdf]

Table S3. Number of genes regulated uniquely or coincidently between different isoforms.

		UP		DOWN		TOTAL	
		No. genes	%	No. genes	%	No. genes	%
Total		533		812		1345	
Unique		443	83	615	76	1058	79
Coincident		90	17	197	24	287	21
Coincidence	2 isoforms	70	13	111	14	181	13
	3 isoforms	16	3	39	5	55	4
	4 isoforms	4	1	34	4	38	3
	5 isoforms	0	0	13	2	13	1

Table S3
