# Supplementary material for: Depletion of Human Histone H1 Variants Uncovers Specific Roles in Gene Expression and Cell Growth
Source: PLoS Genet. 2008 Oct 17;4(10):e1000227. doi: 10.1371/journal.pgen.1000227 (PMC2563032; doi:10.1371/journal.pgen.1000227)
Supplement: Table S4 — Effect of H1 variant inhibition on the expression of 84 genes key to cell cycle regulation. (0.02 MB PDF) [file pgen.1000227.s008.pdf]

Table S4. Effect of H1 variant inhibition on the expression of 84 genes key to cell cycle regulation.

Gene symbol	Control ±Dox	H1.0 sh ±Dox	H1.2 sh ±Dox [b]	H1.4 sh ±Dox
BRCA1	-1.13	1.93	-2.72	-2.34
RAD51	-1.43	1.38	-2.70	-1.85
DDX11	-1.42	-1.97	-2.66	-1.48
CDKN3	-1.29	1.40	-2.41	-2.09
MCM2	-1.03	-1.21	-2.28	-1.78
BIRC5	-1.26	1.67	-2.27	-16.27
CDK2	-1.22	1.28	-2.25	-1.22
RBL1	-1.25	1.21	-2.22	-1.38
CDC2	-1.19	1.57	-2.04	-1.49
MCM5	-1.28	1.17	-2.00	-2.59
MAD2L1	-1.01	1.19	-1.92	-1.51
HERC5	1.36	2.10	-1.91	1.35
CDKN2B	-1.85	-1.66	-1.91	-1.31
MKI67	-1.32	1.82	-1.86	-2.98
CDKN2A	-1.62	-1.22	-1.81	1.05
GTSE1	-1.49	1.42	-1.77	-2.06
PCNA	-1.26	1.42	-1.76	-2.16
CHEK1	-1.20	1.12	-1.68	-1.47
CDC20	-1.21	1.46	-1.62	-1.25
KNTC1	-1.16	1.39	-1.57	-1.67
CCNB2	-1.20	1.13	-1.53	-1.71
GAPDH [a]	1.27	*-1.23*	*-1.52*	-1.02
RAD1	-1.35	2.27	-1.50	-1.77
MCM3	-1.16	1.23	-1.46	-1.31
MRE11A	-1.28	2.04	-1.42	-1.64
BCL2	1.76	2.15	-1.35	-1.23
CKS2	1.23	2.15	-1.31	-1.57
CKS1B	-1.02	1.55	-1.29	-1.07
RPA3	-1.26	1.29	-1.22	-1.60
RBBP8	-1.12	1.39	-1.21	-1.30
CHEK2	1.01	-1.08	-1.19	-1.65
KPNA2	-1.14	1.29	-1.18	-1.07
CDK6	-1.79	1.49	-1.17	-1.07
ACTB [a]	-1.06	*-1.19*	*-1.17*	1.19
SKP2	-1.16	1.20	-1.17	-1.53
ABL1	-1.09	1.27	-1.10	-1.15
CCNF	-1.27	1.49	-1.09	-1.30
CCNB1	-1.21	1.53	-1.07	-1.60
HPRT1 [a]	-1.12	*1.10*	*-1.06*	-1.65
CDK4	-1.04	-1.06	-1.05	-1.02
NBN	-1.71	1.25	-1.01	-2.47
CCNE1	-1.34	-3.24	-1.00	-1.34
MCM4	-1.29	1.99	1.03	-2.47
RAD17	-1.14	1.29	1.06	-1.44
ATM	-1.40	2.04	1.09	-1.31
CUL3	-2.01	1.09	1.11	-1.17
CCNH	-1.27	1.26	1.11	-1.16
RPL13A [a]	-1.02	*1.20*	*1.12*	1.19
GADD45A	1.15	1.32	1.13	1.03
CDKN1B	-1.14	1.83	1.19	-2.05
RB1	-1.36	-1.01	1.19	1.05
CDC16	-1.04	1.13	1.19	-1.09
BCCIP	-1.33	1.30	1.20	-1.20
TP53	1.39	1.13	1.22	1.24
MAD2L2	1.07	1.73	1.24	-1.24
HUS1	-1.28	1.82	1.25	-1.54
CCND1	-1.26	-1.22	1.25	-1.43
UBE1	-1.18	-1.16	1.26	1.21
CDK5RAP1	-1.83	-1.13	1.26	-1.53
CCNT1	-1.16	1.18	1.26	-1.34
E2F4	-1.09	-1.16	1.27	-1.13
CDK7	-1.09	1.50	1.27	1.02
CUL2	-1.12	1.02	1.29	-1.14
CDC34	-1.26	1.39	1.31	-2.06
BAX	-1.16	1.74	1.33	-1.22
RAD9A	-1.03	1.02	1.35	-1.42
CCNC	-1.56	1.01	1.35	1.44
CCNG1	-1.14	-1.09	1.36	1.61
CUL1	-1.09	1.02	1.37	-1.08
CCNG2	-1.18	-1.11	1.39	-1.87
GTF2H1	-1.35	1.71	1.40	-1.62
MNAT1	-1.21	1.32	1.44	-1.05
ANAPC4	-1.37	-1.01	1.48	1.02
ATR	-1.27	1.37	1.49	-1.30
CDK5R1	-1.55	1.22	1.49	1.21
CCNT2	-1.26	1.22	1.58	-1.63
CDKN1A	-1.02	1.52	1.58	1.29
SUMO1	-1.50	1.26	1.63	-1.40
SERTAD1	-1.29	1.01	1.66	-1.09
CDK8	-1.01	1.05	1.69	1.10
B2M [a]	-1.05	*1.11*	*1.69*	1.19
DNM2	-1.27	1.67	1.71	-1.26
ANAPC2	-1.46	-1.01	1.71	-2.11
TFDP1	-2.17	1.22	2.01	-1.63
TFDP2	-1.34	1.34	2.16	1.15
RBL2	-1.24	1.02	2.71	1.13

[a] Five control/housekeeping genes are included in the RT *Profiler* PCR array: B2M, HPRT1, RPL13A, GAPDH and ACTB.
[b] Fold-change in the presence of Dox versus in the absence of Dox (6 days) is presented in ascending order for the H1.2 shRNA cell line.

Table S4
