# Supplementary material for: Depletion of Human Histone H1 Variants Uncovers Specific Roles in Gene Expression and Cell Growth
Source: PLoS Genet. 2008 Oct 17;4(10):e1000227. doi: 10.1371/journal.pgen.1000227 (PMC2563032; doi:10.1371/journal.pgen.1000227)
Supplement: Table S5 — Nomenclature of genes shown in Figure 6. (0.02 MB PDF) [file pgen.1000227.s009.pdf]

Table S5. Nomenclature of genes shown in Figure 6.

Symbol	GenBankNo.	Name
AKT3	NM_005465	V-akt murine thymoma viral oncogene homolog 3 (protein kinase B, gamma)
AURKB	NM_004217	Aurora kinase B
BCL2	NM_000633	B-cell CLL/lymphoma 2
BIRC5	NM_001168	Baculoviral IAP repeat-containing 5 (survivin)
BLM	NM_000057	Bloom syndrome
BRCA2	NM_000059	Breast cancer 2, early onset
C14orf109	BU739864	Chromosome 14 open reading frame 130
C20orf46	NM_018354	Chromosome 20 open reading frame 46
CALR	NM_004343	Calreticulin
CCNB1	NM_031966	Cyclin B1
CCNB2	NM_004701	Cyclin B2
CCNG2	NM_004354	Cyclin G2
CDC2	NM_001786	Cell division cycle 2, G1 to S and G2 to M
CDC20	NM_001255	CDC20 cell division cycle 20 homolog (S. cerevisiae)
CDC23	NM_004661	CDC23 (cell division cycle 23, yeast, homolog)
CDC45L	NM_003504	CDC45 cell division cycle 45-like (S. cerevisiae)
CDC6	NM_001254	CDC6 cell division cycle 6 homolog (S. cerevisiae)
CDKN3	NM_005192	Cyclin-dependent kinase inhibitor 3 (CDK2-associated dual specificity phosphatase)
CKB	NM_001823	Creatine kinase, brain
CKS2	NM_001827	CDC28 protein kinase regulatory subunit 2
CYBASC3	NM_153611	Cytochrome b, ascorbate dependent 3
DIAPH3L	BC041395	Homo sapiens, similar to Diaphanous homolog 3 (Drosophila)
DTL	NM_016448	Denticleless homolog (Drosophila)
EGFP	EMBL_C11	EGFP
ESRRB	NM_004452	Estrogen-related receptor beta
EXO1	NM_130398	Exonuclease 1
FAS	NM_000043	Fas (TNF receptor superfamily, member 6)
FGFR2	NM_023028	Fibroblast growth factor receptor 2
HIST1H1C	NM_005319	Histone 1, H1c / H1.2
HSD11B1	NM_005525	Hydroxysteroid (11-beta) dehydrogenase 1
HSD17B2	NM_002153	Hydroxysteroid (17-beta) dehydrogenase 2
HSD17B8	NM_014234	Hydroxysteroid (17-beta) dehydrogenase 8
HSPB2	NM_001541	Heat shock 27kDa protein 2
HSPCA	NM_005348	Heat shock protein 90kDa alpha (cytosolic), class A member 1
IFIT2	NM_001547	Interferon-induced protein with tetratricopeptide repeats 2
ITGB2	NM_000211	Integrin, beta 2 (complement component 3 receptor 3 and 4 subunit)
KIT	NM_000222	V-kit Hardy-Zuckerman 4 feline sarcoma viral oncogene homolog
KNTC2	NM_006101	Kinetochore associated 2
KRT17	NM_000422	Keratin 17
KRT5	NM_000424	Keratin 5 (epidermolysis bullosa simplex, Dowling-Meara/Kobner/Weber-Cockayne types)
LPXN	NM_004811	Leupaxin
MAD2L1	NM_002358	MAD2 mitotic arrest deficient-like 1 (yeast)
MAP2K4	NM_003010	Mitogen-activated protein kinase kinase 4
MAP4K3	NM_003618	Mitogen-activated protein kinase kinase kinase kinase 3
MCM6	NM_005915	MCM6 minichromosome maintenance deficient 6 (MIS5 homolog, S. pombe)
MELK	NM_014791	Maternal embryonic leucine zipper kinase
NCOA1	NM_147223	Nuclear receptor coactivator 1
NME2	NM_002512	Non-metastatic cells 1, protein (NM23A) expressed in
NRIP1	NM_003489	Nuclear receptor interacting protein 1
NUSAP1	NM_016359	Nucleolar and spindle associated protein 1
PGR	NM_000926	Progesterone receptor
PPARG	NM_015869	Peroxisome proliferative activated receptor, gamma
PRC1	NM_003981	Protein regulator of cytokinesis 1
RBBP8	NM_002894	Retinoblastoma binding protein 8
RFC3	NM_002915	Replication factor C (activator 1) 3, 38kDa
RPL7	NM_000971	Ribosomal protein L7
S100A2	NM_005978	S100 calcium binding protein A2
SART3	NM_014706	Squamous cell carcinoma antigen recognised by T cells 3
SCGB1A1	NM_003357	Secretoglobin, family 1A, member 1 (uteroglobin)
SCGB1C1	NM_145651	Secretoglobin, family 1C, member 1
SERPINB2	NM_002575	Serpin peptidase inhibitor, clade B (ovalbumin), member 2
SHBG	NM_001040	Mannose-P-dolichol utilization defect 1
SMARCA2	NM_003070	SWI/SNF related, matrix associated, actin dependent regulator of chromatin, subfamily a, member 2
SMARCA3	NM_003071	SWI/SNF related, matrix associated, actin dependent regulator of chromatin, subfamily a, member 3
SURB7	NM_004264	SRB7 suppressor of RNA polymerase B homolog (yeast)
TFF1	NM_003225	Trefoil factor 1 (breast cancer, estrogen-inducible sequence expressed in)
TOP2A	NM_001067	Topoisomerase (DNA) II alpha 170kDa
WISP1	NM_003882	WNT1 inducible signaling pathway protein 1
WNT10B	NM_003394	Wingless-type MMTV integration site family, member 10B
XRCC5	NM_021141	X-ray repair complementing defective repair in Chinese hamster cells 5
ZNF533	NM_152520	Zinc finger protein 533
