# Supplementary material for: Depletion of Human Histone H1 Variants Uncovers Specific Roles in Gene Expression and Cell Growth
Source: PLoS Genet. 2008 Oct 17;4(10):e1000227. doi: 10.1371/journal.pgen.1000227 (PMC2563032; doi:10.1371/journal.pgen.1000227)
Supplement: Table S6 — Nomenclature of genes included in the RT Profiler PCR Array. (0.02 MB PDF) [file pgen.1000227.s010.pdf]

Table S6. Nomenclature of genes included in the RT *Profiler* PCR Array

Symbol	GenBankNo.	Name
ABL1	NM_005157	V-abl Abelson murine leukemia viral oncogene homolog 1
ACTB*	NM_001101	Actin, beta
ANAPC2	NM_013366	Anaphase promoting complex subunit 2
ANAPC4	NM_013367	Anaphase promoting complex subunit 4
ATM	NM_000051	Ataxia telangiectasia mutated (includes complementation groups A, C and D)
ATR	NM_001184	Ataxia telangiectasia and Rad3 related
B2M*	NM_004048	Beta-2-microglobulin
BAX	NM_004324	BCL2-associated X protein
BCCIP	NM_016567	BRCA2 and CDKN1A interacting protein
BCL2	NM_000633	B-cell CLL/lymphoma 2
BIRC5	NM_001168	Baculoviral IAP repeat-containing 5 (survivin)
BRCA1	NM_007294	Breast cancer 1, early onset
BRCA2	NM_000059	Breast cancer 2, early onset
CCNB1	NM_031966	Cyclin B1
CCNB2	NM_004701	Cyclin B2
CCNC	NM_005190	Cyclin C
CCND1	NM_053056	Cyclin D1
CCND2	NM_001759	Cyclin D2
CCNE1	NM_001238	Cyclin E1
CCNF	NM_001761	Cyclin F
CCNG1	NM_004060	Cyclin G1
CCNG2	NM_004354	Cyclin G2
CCNH	NM_001239	Cyclin H
CCNT1	NM_001240	Cyclin T1
CCNT2	NM_001241	Cyclin T2
CDC16	NM_003903	Cell division cycle 16 homolog (S. cerevisiae)
CDC2	NM_001786	Cell division cycle 2, G1 to S and G2 to M
CDC20	NM_001255	Cell division cycle 20 homolog (S. cerevisiae)
CDC34	NM_004359	Cell division cycle 34 homolog (S. cerevisiae)
CDK2	NM_001798	Cyclin-dependent kinase 2
CDK4	NM_000075	Cyclin-dependent kinase 4
CDK5R1	NM_003885	Cyclin-dependent kinase 5, regulatory subunit 1 (p35)
CDK5RAP1	NM_016408	CDK5 regulatory subunit associated protein 1
CDK6	NM_001259	Cyclin-dependent kinase 6
CDK7	NM_001799	Cyclin-dependent kinase 7 (MO15 homolog, Xenopus laevis, cdk-activating kinase)
CDK8	NM_001260	Cyclin-dependent kinase 8
CDKN1A	NM_000389	Cyclin-dependent kinase inhibitor 1A (p21, Cip1)
CDKN1B	NM_004064	Cyclin-dependent kinase inhibitor 1B (p27, Kip1)
CDKN2A	NM_000077	Cyclin-dependent kinase inhibitor 2A (melanoma, p16, inhibits CDK4)
CDKN2B	NM_004936	Cyclin-dependent kinase inhibitor 2B (p15, inhibits CDK4)
CDKN3	NM_005192	Cyclin-dependent kinase inhibitor 3 (CDK2-associated dual specificity phosphatase)
CHEK1	NM_001274	CHK1 checkpoint homolog (S. pombe)
CHEK2	NM_007194	CHK2 checkpoint homolog (S. pombe)
CKS1B	NM_001826	CDC28 protein kinase regulatory subunit 1B
CKS2	NM_001827	CDC28 protein kinase regulatory subunit 2
CUL1	NM_003592	Cullin 1
CUL2	NM_003591	Cullin 2
CUL3	NM_003590	Cullin 3
DDX11	NM_004399	DEAD/H (Asp-Glu-Ala-Asp/His) box polypeptide 11 (CHL1-like helicase homolog, S. cerevisiae)
DIRAS3	NM_004675	DIRAS family, GTP-binding RAS-like 3
DNM2	NM_004945	Dynamin 2
E2F4	NM_001950	E2F transcription factor 4, p107/p130-binding
GADD45A	NM_001924	Growth arrest and DNA-damage-inducible, alpha
GAPDH*	NM_002046	Glyceraldehyde-3-phosphate dehydrogenase
GTF2H1	NM_005316	General transcription factor IIH, polypeptide 1, 62kDa
GTSE1	NM_016426	G-2 and S-phase expressed 1
HERC5	NM_016323	Hect domain and RLD 5
HGDC	SA_00105	Human Genomic DNA Contamination
HPRT1*	NM_000194	Hypoxanthine phosphoribosyltransferase 1 (Lesch-Nyhan syndrome)
HUS1	NM_004507	HUS1 checkpoint homolog (S. pombe)
KNTC1	NM_014708	Kinetochore associated 1
KPNA2	NM_002266	Karyopherin alpha 2 (RAG cohort 1, importin alpha 1)
MAD2L1	NM_002358	MAD2 mitotic arrest deficient-like 1 (yeast)
MAD2L2	NM_006341	MAD2 mitotic arrest deficient-like 2 (yeast)
MCM2	NM_004526	MCM2 minichromosome maintenance deficient 2, mitotin (S. cerevisiae)
MCM3	NM_002388	MCM3 minichromosome maintenance deficient 3 (S. cerevisiae)
MCM4	NM_005914	MCM4 minichromosome maintenance deficient 4 (S. cerevisiae)
MCM5	NM_006739	MCM5 minichromosome maintenance deficient 5, cell division cycle 46 (S. cerevisiae)
MKI67	NM_002417	Antigen identified by monoclonal antibody Ki-67
MNAT1	NM_002431	Menage a trois homolog 1, cyclin H assembly factor (Xenopus laevis)
MRE11A	NM_005590	MRE11 meiotic recombination 11 homolog A (S. cerevisiae)
