# Supplementary material for: Depletion of Human Histone H1 Variants Uncovers Specific Roles in Gene Expression and Cell Growth
Source: PLoS Genet. 2008 Oct 17;4(10):e1000227. doi: 10.1371/journal.pgen.1000227 (PMC2563032; doi:10.1371/journal.pgen.1000227)
Supplement: Text S1 — Suppplementary figure legends and Materials & Methods. (0.12 MB PDF) [file pgen.1000227.s011.pdf]

SUPPLEMENTARY INFORMATION

SUPPLEMENTARY FIGURE LEGENDS

Figure S1. Characterization of human histone H1 variant-specific antibodies and analysis of the H1 variants pattern in T47D cells.

(A) Alignment of N-terminal sequences of human H1 variants underlying peptide sequences used for production of polyclonal antibodies.

(B) Newly-generated antibodies against H1.1, 2, 3 and 5 recognize specifically its corresponding variant in a Western blot with recombinant human H1 variants produced in yeast (courtesy of N. Happel and D. Doenecke). H1.4 antibody does not recognize its histone. A commercial H1.0 antibody (Abcam ab11079) is also highly specific. Commercial histone H1 (phospho T146) antibody (Abcam ab3596) uniquely recognizes recombinant H1.4 variant produced in yeast.

(C) Western blot with the H1 phospho-T146 antibody on recombinant H1.4 produced in bacteria (lane 1), total H1 extracted from T47D cells untreated (lane 2) or treated with colcemid (50 ng/ml) over-night (lane 3).

(D) SDS-PAGE of H1 extracted from exponentially growing T47D cells, stained with Coomassie. Bands corresponding to H1 variants are labeled. Quantification of bands using Image Gauge (Fujifilm) software indicated that H1.0 represents 9.5% of total H1, H1.2 accounts for 23.5%, and the remaining 67% contains H1.3, H1.4 and H1.5.

(E) T47D and HeLa H1.2 KD cells were untreated or Dox-treated for 6 days. Total H1 was extracted and resolved on SDS-PAGE. After Coomassie staining, H1 bands were quantified densitometrically. Total H1 was reduced approximately 20% in T47D and 28% in HeLa H1.2 KD.

(F) Separation of H1 variants by reverse phase-FPLC and recognition by specific antibodies. Samples obtained after H1 purification procedure were further purified in a FPLC system using an Amersham Resource RPC 1ml column in buffer A (0.065% trifluoroacetic acid (TFA), 2% acetonitrile acid (ACN) in water) with a linear gradient from 41% to 44.5% of buffer B (0.05% TFA, 80% ACN). The elution

profile was constructed from absorbance measurements at 210 nm. Purified samples were dried in a speedvac, dissolved in water, and submitted to Western blot with antibodies generated in-house.

(G) Coomassie staining of recombinant, bacterially-produced H1 variant obtained from Alexis Biochemicals (500 ng).

(H) Total H1 was extracted from T47D H1 variant KD ±Dox cells and loaded (1μg protein for H1.5 and 3μg for the other H1 KD) on a 12% SDS-PAGE, together with serial dilutions (150 to 12.5ng) of each correspondent recombinant H1 variant, for immunoblotting with variant-specific antibodies. Comparison of signal intensity of H1 preparations with the standard curve generated with serially-diluted recombinant H1 allowed to determine the proportion of each variant in the total H1 content.

Figure S2. **Rescue of the deleterious effect of H1.4 shRNA by transient expression of recombinant shRNA-resistant H1.4.** T47D H1.4 KD cells were grown in the absence or presence of Dox. At day 3, pCDN4-based plasmid expressing HA-tagged, shRNA-resistant H1.4 was transfected with Lipofectamine Plus (Invitrogen) and Zeocin was added for selection. Live cells were observed with an inverted microscope at day 6 **(A)**, or at day 9 after staining with crystal violet **(B)**.

Figure S3. **Incorporation of recombinant HA-tagged H1 isoforms into chromatin.**

(A) Western blot with an anti-HA antibody of chromatin samples obtained from T47D cells stably expressing the HA-tagged H1 variants indicated.

(B) Immunoflaresce analysis of T47D cells stably expressing HA-tagged H1.2 histone.

(C) ChIP analysis with an anti-HA antibody of the presence of several HA-tagged H1 isoforms at the MMTV promoter or ß-globin gene. Enrichment over material immunoprecipitated with preimmune IgG is indicative of specific presence of the tagged H1 variant.

Figure S4. **Cell cycle gene alterations in H1.2 knock-down cells.**

(A) Expression of several cell cycle regulation genes indicated was measured by RT-qPCR with specific oligonucelotides in H1.2 KD and control cells treated or not with Dox for 6 days. GAPDH expression was measured for normalization. Data is expressed as relative units specific gene/GAPDH. The values represent the mean and SD of a representative experiment performed in triplicate.

(B) Cell cycle gene expression changes in H1.2 knock-down cells along time after Dox addition. H1.2 KD cells growing in 10% serum were treated with Dox for the time points indicated and RNA was extracted. Gene expression was measured by RT-quantitative PCR with specific oligonucleotides.

(C) H1.2 KD cells grown for six days in the presence or absence of Dox and without serum for the last two days, were treated with serum and RNA was extracted at time points indicated for gene expression analysis by RT-qPCR.

SUPPLEMENTARY MATERIALS AND METHODS

Customized microarray hybridization and data analysis

A cDNA microarray platform containing 826 cDNA clones that were selected for its involvement in breast cancer was generated (B. Miñana, L. Sumoy, M. Beato, A. Jordan, C. Ballare, M. Melia; GEO accession number GPL5953; http://www.ncbi.nlm.nih.gov/projects/geo/index.cgi). cDNA inserts were PCR amplified and spotted on Corning UltraGAPS amino-modified glass slides. mRNA samples were processed for first and second strand cDNA synthesis and in vitro transcription with T7 RNA polymerase, basically as reported elsewhere [1, 2]. Universal reference RNA was obtained from Statagene. RNA was directly labeled with Cy3- or Cy5-dUTP (Amersham) and hybridized to spotted slides as described [3]. After washing, fluorescent images were obtained using a G2565BA Microarray Scanner System (Agilent) and TIFF images were quantified using GenePix 6.0 (Molecular Devices) software.

Raw data was processed using MARGE, an in house developed web implementation of LIMMA, a microarray statistical analysis package of Bioconductor (http://www.bioconductor.org) that is run in the R programming environment [4-6]. Gene intensities were background subtracted (taking mean of channel intensities and median of background). Spots with intensities <2 times the local background in either or both dye filter channels (Cy3 or Cy5) as well as controls were excluded from normalization, and were referred as “not reliable”. An intensity dependent normalization algorithm (global lowess) was applied using a smoothing factor f=0.2 for all experiments. Normalized Log_2Ratios (Intensity Cy5/Intensity Cy3) were scaled so that they all had the same median absolute standard deviation across all the arrays, to give the same weight to each gene, and not only due to the magnitude of the expression ratio [7]. The computed B statistic rank value from all replicate hybridizations was used to determine the genes with significant changes. We considered genes that showed a 1.4-fold gene up or down-regulation relative to control sample with a B-rank value above the 90th percentile as significant. The value of fold change or copy number relative change

was calculated as $2^{Log2Ratio}$, if the value of the ratio was >0, or $2^{-1/Log2Ratio}$, if it was <0. In order to do the statistical analysis of the data, we have used the open-source, freely available software package for microarray data management and analysis TM4 obtained from TIGR (http://www.tigr.org/software/) [8] that applies the Significance Analyses of Microarrays (SAM) method.

Illumina microarray hybridization and data analysis

For genome-wide microarray experiments, an Illumina platform was used: Platform: GPL6104 Illumina humanRef-8 v2.0 expression beadchip.

For each sample 200ng of total RNA were reverse transcribed, amplified by in vitro transcription and labelled with biotin-UTP using the Illumina Total Prep RNA amplification kit (IL1791, Ambion) following the manufacturer's instructions. Labelled sample quality was assessed by spectrophotometry and bioanalyzer. After preheating at 65ºC for 5 min.

For each sample, 750ng of biotinylated cRNA were hybridized in a BeadChip Hyb Chamber with rocking 16 h at 58ºC. The day after the bead arrays were washed with Illumina proprietary washing solutions in a Hybex waterbath: first with static incubation for 10 min at 55ºC in E1BC solution, followed by 10 rinses by dipping in the same solution and shaking 5 min at 90 rpm in an orbital shaker; the next wash was by dipping 10 times in 100% ethanol and shaking 10 min at 110 rpm in an orbital shaker; this was followed by another wash in E1BC solution with 10 dippings followed by 2 min shaking at 90 rpm. Washed bead arrays were blocked in E1 buffer 10 min in rocking incubator and 10 additional minutes with 2 ml of E1 buffer plus streptavidin-Cy3. The fluorescent reagent was washed away with E1BC solution with 10 dippings plus 5 min shaking at 140 rpm. Finally beadarrays were dried by centrifugation 4 min at 275rcf, followed by scanning in a Illumina Beadstation.

The Beadscan software was used to control de scanner, generate .tif images and extract the raw data as tabulated text files. Default settings for DirectHyb Gene Expression were used.

The raw data was summarized per probe using BeadStudio software Gene Expression module and the summary data file was processed using the PILLA web einterface tool (Lozano et al, unpublished), an implementation of the Lumi package [9] developed within the Bioconductor project in the R statistical programming environment [5]. Data were normalized using the rsn method and vst as the variance stabilization method. The log2 intensities were median centered and log ratios were computed as differences in log2 intensities for each probe. The SAM (significance analysis of microarrays) two class unpaired comparison test was applied with 100 permutations to detect statistically significant differences in gene expression between treated and control conditions [10].

All microarray hybridizations were performed at the Microarrays unit of the Centre de Regulació Genòmica (CRG).

SUPPLEMENTARY REFERENCES

1. Eberwine J, Yeh H, Miyashiro K, Cao Y, Nair S, et al. (1992) Analysis of gene expression in single live neurons. Proc Natl Acad Sci U S A 89(7): 3010-3014.

2. VanGelder RN, vonZastrow ME, Yool A, Dement WC, Barchas JD, et al. (1990) Amplified RNA synthesized from limited quantities of heterogeneous cDNA. Proc Natl Acad Sci U S A 87(5): 1663-1667.

3. Richter A, Schwager C, Hentze S, Ansorge W, Hentze MW, et al. (2002) Comparison of fluorescent tag DNA labeling methods used for expression analysis by DNA microarrays. BioTechniques 33(3): 620-8, 630.

4. Dudoit S, Gentleman RC, Quackenbush J. (2003) Open source software for the analysis of microarray data. BioTechniques Suppl: 45-51.

5. Gentleman RC, Carey VJ, Bates DM, Bolstad B, Dettling M, et al. (2004) Bioconductor: open software development for computational biology and bioinformatics. Genome Biol 5(10): R80.

6. Wettenhall JM, Smyth GK. (2004) limmaGUI: a graphical user interface for linear modeling of microarray data. Bioinformatics 20(18): 3705-3706.

7. Yang YH, Dudoit S, Luu P, Lin DM, Peng V, et al. (2002) Normalization for cDNA microarray data: a robust composite method addressing single and multiple slide systematic variation. Nucleic Acids Res 30(4): e15.

8. Saeed AI, Sharov V, White J, Li J, Liang W, et al. (2003) TM4: a free, open-source system for microarray data management and analysis. BioTechniques 34(2): 374-378.

9. Du P, Kibbe WA, Lin SM. (2008) lumi: a pipeline for processing Illumina microarray. Bioinformatics 24(13): 1547-1548.

10. Tusher VG, Tibshirani R, Chu G. (2001) Significance analysis of microarrays applied to the ionizing radiation response. Proc Natl Acad Sci U S A 98(9): 5116-5121.
